# Breaking the Cycle of Marginalization: How to Involve Local Communities in Multi-stakeholder Initiatives?

**DOI:** 10.1007/s10551-022-05252-5

**Published:** 2022-09-22

**Authors:** Manon Eikelenboom, Thomas B. Long

**Affiliations:** 1 Faculty of Social Sciences, Vrije Universiteit Amsterdam, De Boelelaan 1105, 1081 HV Amsterdam, The Netherlands; 2 University of Groningen, Campus Fryslân, Wirdumerdijk 34, 8911 CE Leeuwarden, The Netherlands

**Keywords:** Multi-stakeholder initiatives, Community involvement, Action research

## Abstract

While the benefits of including local communities in multi-stakeholder initiatives have been acknowledged, their successful involvement remains a challenging process. Research has shown that large business interests are regularly over-represented and that local communities remain marginalized in the process. Additionally, little is known about how procedural fairness and inclusion can be managed and maintained during multi-stakeholder initiatives. The aim of this study was therefore to investigate how marginalized stakeholders, and local communities in particular, can be successfully involved during the course of a multi-stakeholder initiative. An action research approach was adopted where the first author collaborated with a social housing association on an initiative to involve the local community in the design and implementation of circular economy approaches in a low-income neighbourhood. This study contributes to the multi-stakeholder initiative literature by showing that the successful involvement of marginalized stakeholders requires the initiators to continuously manage a balance between uncertainty–certainty, disagreement–agreement and consensus- and domination-based management strategies. Furthermore, our study highlights that factors which are regularly treated as challenges, including uncertainty and disagreement, can actually play a beneficial role in multi-stakeholder initiatives, emphasizing the need to take a temporally sensitive approach. This study also contributes to the circular economy literature by showing how communities can play a bigger role than merely being consumers, leading to the inclusion of a socially oriented perspective which has not been recognized in the previous literature.

**Supplementary Information:**

The online version contains supplementary material available at 10.1007/s10551-022-05252-5.

## Introduction

Multi-stakeholder initiatives are increasingly used to address social and ecological problems (De Bakker et al., 2019). In these initiatives, actors from business, civil society and governmental institutions come together to find a common approach to an issue that affects them all and that is too complex to be addressed effectively without collaboration (Baumann-Pauly et al., 2017; Roloff, 2008). By engaging in multi-stakeholder initiatives, businesses, governments and civil society organizations can address their ethical responsibilities in relation to inclusivity by incorporating diverse societal perspectives and creating economic, social and environmental benefits (MacDonald et al., 2019). These initiatives can for example help organizations contribute to their community by addressing local economic, social and environmental concerns (De Bakker et al., 2019). Examples of multi-stakeholder initiatives include the Fair Labor Association and Fair Wear Foundation, in which businesses, governments, knowledge institutions and NGOs collaborate to develop better labour conditions and fairer ways of manufacturing clothes. 

While local communities are not always included in multi-stakeholder initiatives, their inclusion can increase the legitimacy of decision-making processes and enhance the long-term viability and benefits of initiatives (Lu et al., 2018). There are multiple different definitions of community. Nevertheless, scholars generally agree that communities can be characterized by three factors: geography, interaction and identity (Dunham et al., 2006). In this paper, we characterize communities by geography or as a ‘community of place’, referring to local community as consisting of both individual citizens and groups of citizens organized to present their shared interests, residing within the same geographic region (Bowen et al., 2010; Dunham et al., 2006). The involvement of these actors in multi-stakeholder initiatives can enable improved outcomes. The ‘Grainger Town Project’, a collaboration between businesses, local government and citizens to restore the historic city centre of Newcastle, for example shows how involving local communities can help to successfully address their needs (Roloff, 2008). Other benefits of involving local communities in multi-stakeholder initiatives include the legitimization of decision-making processes, joint learning and sense-making, and the enhanced acceptance of outcomes (Baumann-Pauly et al., 2017; Mena & Palazzo, 2012). For example, it has been argued that the Forest Stewardship Council, which aims to protect forests globally, has induced more social change compared to the similar Sustainable Forestry Initiative, due to its regular consultations with local communities and extensive stakeholder meetings (Cubbage & Moore, 2008).

While the legitimacy of local communities’ claims has been acknowledged, the involvement of these actors in multi-stakeholder initiatives remains a challenging process (Fransen & Kolk, 2007; Lu et al., 2018). Therefore, local communities, especially those in vulnerable positions, including minority groups and those living in poverty, are often marginalized in the process, meaning that they lack voice and power (Derry, 2012). Multi-stakeholder research has shown that both inclusion and procedural fairness are of central importance for the involvement of marginalized stakeholders (Baumann-Pauly et al., 2017; Boström, 2006; Fransen & Kolk, 2007). However, research findings have also highlighted that most multi-stakeholder initiatives fail to achieve either of these elements (Cheyns & Riisgaard, 2014; Easter et al., 2022; Fransen & Kolk, 2007). Research has for example indicated that most multi-stakeholder initiatives exhibit a lack of inclusiveness and that large company interests are over-represented (Baumann-Pauly et al., 2017; Dentoni et al., 2018; Fougère & Solitander, 2020). NGOs are regularly included as a token for civil society representation; however, the outcomes of NGO inclusion are not necessarily beneficial to local communities as NGOs also pursue self-interested objectives (Banerjee, 2014). Furthermore, when local communities are involved, they do not always have influence on the decisions made and therefore remain marginalized in the process (Easter et al., 2022; Mena & Palazzo, 2012). Several challenges inhibit the involvement of local communities in multi-stakeholder initiatives, including its time-consuming nature, the use of the language of dominant parties, the limited power of local communities and a lack of knowledge within local communities (Edmunds & Wollenberg, 2002; Khazaei et al., 2015). To enable local communities to become active and equal participants in multi-stakeholder initiatives, increased attention must be given to how their involvement can be managed. This may include acquiring a deep understanding of local community perspectives and improving their knowledge and confidence (Khazaei et al., 2015).

While the literature has provided relevant insights into how diverse challenges can be managed in multi-stakeholder initiatives (Gray & Purdy, 2018), these efforts have been mostly directed to conflicts concerning consensus building among central actors, neglecting challenges related to the involvement of marginalized stakeholders. Additionally, while some initial guidelines for the inclusion of marginalized stakeholders at the start of initiatives have been provided, such as assigning these stakeholders a clear role (Fransen & Kolk, 2007), most guidelines fail to address how to manage and maintain marginalized stakeholder inclusion during the course of multi-stakeholder initiatives. This is of central importance, as research has shown that maintaining inclusiveness and procedural fairness can be difficult due to the strategic actions of stakeholders who wish to evade certain ethical issues or because marginalized stakeholder groups opt out of initiatives (Fransen & Kolk, 2007; Moog et al., 2015). Furthermore, most previous studies have focussed on large multi-stakeholder initiatives in which companies and international NGOs are central actors, and where local communities have only served peripheral roles (Fransen & Kolk, 2007; Gray & Purdy, 2018). To address these gaps in the literature this research seeks to answer the following question: how can marginalized stakeholders, and local communities in particular, be successfully involved during the course of a multi-stakeholder initiative? By successful, we refer to the inclusion of relevant local community actors throughout the initiative, as well as the creation and maintenance of procedural fairness, enabling local communities to be active and equal participants in the decision-making process. We explore this question through a process perspective (Roloff, 2008), investigating the involvement of local communities in different phases of a multi-stakeholder initiative and exploring the challenges that were encountered during this process including how these challenges were managed. 

We adopted an action research approach (Susman & Everd, 1978) to investigate the research question. The first author actively participated in the design and execution of an initiative where local communities, next to other stakeholders, were involved in the design and implementation of circular economy approaches in a low-income neighbourhood in the Netherlands. The circular economy—an economic system that replaces the ‘end-of-life’ concept with reducing, reusing, recycling and recovering materials (Kirchherr et al., 2017)—provides an interesting context for this research as multi-stakeholder initiatives, and the involvement of communities in these initiatives, can help address its ethical implications. The circular economy is a response to ethical issues in the linear take-make-dispose system, enabling economic prosperity without compromising the abilities of future generations to meet their needs (Kirchherr et al., 2017). However, researchers have argued that the circular economy itself is not a neutral system and involves multiple ethical considerations (Inigo & Blok, 2019; Murray et al., 2017). First, the circular economy has significant consequences for social equality (Murray et al., 2017) bringing prosperity and a socially positive footprint, but potentially (Mavropoulos & Nilsen, 2020) unintended consequences for some stakeholders (Inigo & Blok, 2019; Mavropoulos & Nilsen, 2020). For example, house sharing initiatives, such as Airbnb, may lead to significant pressures on the housing market, increasing prices, and disturbing local practices (Lee, 2016). Second, circular economy approaches generally involve different actors with different values, interests and priorities, which may lead to unethical behaviours when some actors are systematically favoured over others (Mavropoulos & Nilsen, 2020; Payne & Calton, 2002). Involving local communities in multi-stakeholder initiatives for the circular economy may help to address these ethical issues (Eikelenboom et al., 2021; Lu et al., 2018).

Our results improve our understanding of the successful involvement of marginalized stakeholders, and local communities in particular, in multi-stakeholder initiatives in several ways (Baumann-Pauly et al., 2017; Cheyns & Riisgaard, 2014; Easter et al., 2022; Fransen & Kolk, 2007). First, our study showed that the successful involvement of marginalized stakeholders requires a continuous management of three factors, including uncertainty, disagreement and consensus- vs. domination-based management strategies. Second, our results indicated that factors which are regularly treated as challenges, including uncertainty and disagreement, can actually play a beneficial role in multi-stakeholder initiatives, for example, by enabling the inclusion of unexpected community perspectives. Third, our findings highlighted the importance of combining both consensus- and domination-based strategies to manage multi-stakeholder initiatives in order to reduce inequality and simultaneously allow for the development of new ideas and relationships. In conclusion, our findings highlight that by carefully balancing uncertainty–certainty, disagreement–agreement and domination- and consensus-based management during all stages of the initiative by taking a temporally sensitive approach, marginalized stakeholder inclusion can be improved. Finally, our study also contributes to the circular economy literature (Geissdoerfer et al., 2017; Inigo & Blok, 2019; Kristensen & Mosgaard, 2020; Murray et al., 2017) by showing that local communities can act as co-creators of circular strategies, leading to the inclusion of a socially oriented perspective which has not been recognised in the previous literature.

## Literature

### Multi-stakeholder Initiatives

Researchers have argued that organizations who use traditional types of stakeholder management (e.g. Freeman, 1984; Mitchell et al., 1997) tend to overlook stakeholders who are affected by the organization in favour of those who can affect it (Roloff, 2008). Therefore, new ways of dealing with diverse stakeholder perspectives have been proposed in the form of multi-stakeholder initiatives. Multi-stakeholder initiatives can assume many different forms including policy dialogues, co-management of natural resources and transnational networks among others (Gray & Purdy, 2018). In this paper, we take a broad view on multi-stakeholder initiatives, including initiatives (e.g. Baumann-Pauly et al., 2017), networks (e.g. Roloff, 2008) and partnerships (e.g. Dentoni et al., 2018). Furthermore, we see these initiatives as including both well-known global partnerships and certifications networks that create non-governmental governance mechanisms, as well as smaller projects that focus on fostering dialogues and where local stakeholders participate in decision-making concerning a particular socio-ecological system (Baumann-Pauly et al., 2017; Roloff, 2008; Zimmermann et al., 2021).

Multi-stakeholder initiatives aim to consider all stakeholders as equally important and attempt to engage them in a mutual learning process (Khazaei et al., 2015). In these initiatives, different stakeholders, including businesses, governments and civil society organizations, come together to address issues by communication and collaboration, instead of focussing on one organization and its objectives as the focal point (Baumann-Pauly et al., 2017; Roloff, 2008). The term stakeholder in this context is defined as *any group or individual who can affect or is affected by the approach to the issue addressed by the initiative* (Roloff, 2008, p. 238). Collaboration in multi-stakeholder initiatives is seen as a process that engages a group of interdependent stakeholders with interests in a problem or issue in an interactive deliberation using shared rules, norms, and structures, to share information and/or take coordinated action (Wood & Gray, 1991, p. 11). The objective of collaboration is to create a richer and more comprehensive appreciation of the issue than any of the individual stakeholders could construct alone by viewing it from the perspectives of all involved stakeholders (Baumann-Pauly et al., 2017; Gray & Purdy, 2018). 

**Fig. 1:**
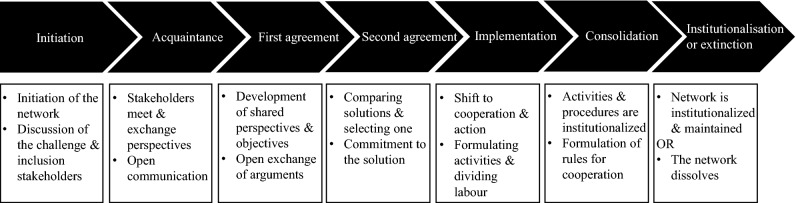
The phases of issue-focussed stakeholder management (based on Roloff, 2008)

Managing multi-stakeholder initiatives is complex due to the wide range of actors, perspectives, values and beliefs involved and can therefore not be managed by one actor alone. Roloff (2008) suggests that organizations should in this case adopt an issue-focussed stakeholder management approach. This approach gives special attention to open interactions and the generation of shared perspectives. The early involvement of different stakeholders is therefore vital, which can assist stakeholders in grasping the complexity of an issue and learning about stakeholder interdependencies (Roloff, 2008). Issue-focussed stakeholder management involves several phases (Fig. 1) through which the perspectives, resources and competencies of different stakeholders can be recombined, resulting in the generation of shared perspectives and solutions (Roloff, 2008).

### Multi-stakeholder Initiatives and Marginalized Stakeholders

It has been argued that the involvement of marginalized stakeholders in multi-stakeholder initiatives is important for several reasons. First, when various interest groups are involved, multi-stakeholder initiatives have the benefit of inclusiveness, meaning that all parties relevant to a specific issue have a say (Boström, 2006). Second, when all participating stakeholders are considered as equal participants in the decision-making process, multi-stakeholder initiatives can also benefit from procedural fairness (Baumann-Pauly et al., 2017). Third, the inclusion of stakeholders with diverse backgrounds can facilitate knowledge sharing and learning between, among and inside the different involved organizations (Fransen & Kolk, 2007; Moog et al., 2015; Palazzo & Scherer, 2006). However, researchers have argued that achieving inclusiveness and procedural fairness, especially of marginalized stakeholder groups, in multi-stakeholder initiatives can be challenging and is often not achieved (Moog et al., 2015). This has several reasons.

First, it is not always clear what constitutes a ‘good’ or ‘appropriate’ stakeholder and what ‘good’ or ‘appropriate’ stakeholder involvement actually entails (Fransen & Kolk, 2007). Fransen and Kolk (2007) point out that stakeholder involvement can mean different things, depending on the type of interaction including for example talking to stakeholder representatives at a conference versus giving stakeholders a clear role. The authors construct a continuum of stakeholder involvement ranging from involvement (broad inclusiveness) to consultation (narrow inclusiveness). While both involvement and consultation can be part of multi-stakeholder initiatives, the differences are notable in terms of the position of particular groups in the process (Fransen & Kolk, 2007). Actors may for instance keep the involvement of marginalized stakeholder groups at a more abstract policy level without giving these groups access to concrete and potentially sensitive information (Fransen & Kolk, 2007).

Second, most multi-stakeholder initiatives do not exhibit the degree of stakeholder inclusion that might be anticipated, in particular regarding the inclusion of the interests and perspectives of marginalized stakeholders (Baumann-Pauly et al., 2017; Cheyns & Riisgaard, 2014; Fransen & Kolk, 2007; Moog et al., 2015). Studies have shown that commercial interests and large international organizations are consistently over-represented, while smaller groups representing minority concerns and interests are systematically under-represented (Fransen & Kolk, 2007; Moog et al., 2015). This lack of inclusiveness can be caused by the strategic action of stakeholders who wish to evade a certain ethical issue instead of actively dealing with it. This is achieved by creating a stakeholder environment that seems credible, but that is of marginal relevance to the topic at hand or by picking and choosing stakeholders according to their willingness to compromise (Fransen & Kolk, 2007). A lack of inclusiveness can also be the result of stakeholder groups opting out of the initiative, leaving willing stakeholder groups only (Moog et al., 2015). Participation in multi-stakeholder initiatives demands significant resources and capacity, which can impose a burden on civic groups’ organizational resources. In addition, participation in multi-stakeholder initiatives can cause serious legitimacy threats to groups due to potential compromises or poor outcomes (Moog et al., 2015). 

Third, even when marginalized stakeholders are included, they are not always able to have an equal voice in the multi-stakeholder initiative (Easter et al., 2022). Decision-making power is wielded by stakeholders on the board, which means that marginalized stakeholders often rely on indirect representation via NGO board participants (Baumann-Pauly et al., 2017). Furthermore, critics have claimed that multi-stakeholder initiatives are extremely limited as spaces of political contestation (Banerjee, 2008; Edward & Willmott, 2008). Moog et al. (2015) argue, for example, that multi-stakeholder initiatives can undermine the ability of concerned citizens to effectively politicize underlying conflicts, as minority perspectives, more radical ethical claims and critiques are regularly excluded or channelled into restricted arenas of well-mannered deliberation. Critics also point to power relations in multi-stakeholder initiatives, arguing that these initiatives serve the interests of dominant corporations at the expense of other stakeholders (Banerjee, 2008). Traditional categories of power linked to economic resources and actor strategies are still at play in multi-stakeholder initiatives, for example, by favouring certain forms of knowledge (e.g. technical, commercial, scientific, expert knowledge) and modes of engagement over others (Cheyns & Riisgaard, 2014). Powerful stakeholders may also withhold effort and information, exclude others from participating and reduce the diversity of perspectives in order to impose their will over others, retain their power and protect their interests (Gray & Purdy, 2014, p. 213). Furthermore, if stakeholders feel that other stakeholders have more power to influence the process, they may feel voiceless, and distrust may lead to their refusal to join the initiative (Huxham & Vangen, 2005). Power issues can also be more subtle, such as when stakeholders are not organized in a way that allows them to fully participate, or when the interests of some stakeholders are not noticed or acknowledged (Gray & Purdy, 2018). In this way, the views of dominant actors may be privileged or reinforced in multi-stakeholder initiatives, reproducing dominant ideologies and conservative forms of common sense (Prem, 2021). The consensus reached conceals asymmetries of power and structural inequalities, exposing disadvantaged groups to greater manipulation and control (Cheyns & Riisgaard, 2014; Prem, 2021). 

Fourth, when diverse stakeholders are included, collaboration and the generation of shared perspectives can be problematic leading to a lack of implementation of potential solutions (Easter et al., 2022; Hovring et al., 2018; Reypens et al., 2019). Consensus is not easily achieved, which is most apparent in the start-up phase of a multi-stakeholder initiative (Zeyen et al., 2016). Even when stakeholders show a strong commitment to collaboration, conflicts may still arise, including conflicts over their relationships, values and the process used to search for agreement (Gray & Purdy, 2018). Such conflicts are especially likely to occur when marginalized stakeholders who hold relatively less power compared to other stakeholders are involved (Matos & Silvestre, 2012). Chávez and Bernal (2008) showed, for example, in their study on public participation in the construction of a hydroelectric power plant in Mexico, that while there was community support for the project, conflict over the scope of the project, compensation to landowners and ownership rights for natural resources surfaced as the project progressed. Gray and Purdy (2018) provide an extensive overview of the reasons for such conflicts in multi-stakeholder initiatives. These include history (stakeholders being at odds for years), distrust, differing interpretations or frames about issues and problems, mandated collaboration, value conflicts and identity differences, differences in risk perception, resource constraints and power differences. Gray and Purdy (2018) also show that more deeply rooted institutional logics can drive conflict, such as when stakeholders originate from diverse societal sectors. Stagnation, resulting from these conflicts, may lead to implementation challenges because members are continuously discussing the purpose, strategy and operations of the particularities of the initiative (Easter et al., 2022; Zeyen et al., 2016). Furthermore, when conflicts go unresolved, multi-stakeholder initiatives may fall apart and stakeholders may abandon their shared vision and adopt individual strategies that block or reverse the initiative (Gray & Purdy, 2018).

Several tactics to address conflicts in multi-stakeholder initiatives and keep these initiatives on track have been proposed. Third parties may for instance conduct conflict assessments to understand the history of conflicts, learn the positions and interests of the involved stakeholders and diagnose the feasibility of a consensus-building process (Gray & Purdy, 2018). Another tactic is acknowledging the involved stakeholders’ critical identities to minimize feelings that these may be threatened by collaboration. Conflict may also be addressed through the identification of leaders who can help stakeholders focus their attention on key issues, create a sense of urgency and persuade stakeholders to collaborate (Gray & Purdy, 2018). When power differences exist, an important strategy is ‘levelling the playing field’ where the focus is on increasing the voice of low power stakeholders and increasing trust (Gray & Purdy, 2018; Purdy, 2012). Furthermore, stakeholders may overcome conflict by exploring each other’s frames which may enable misconceptions about stakeholders’ interests to be overcome and shared frames to be discovered. Reypens et al. (2019) show that domination-based strategies may be necessary in addition to consensus-based strategies usually emphasized in issue-focussed stakeholder management. Within these strategies core stakeholders set the collaborative agenda, recruit partners and steer relationships (Reypens et al., 2019).

In conclusion, while it has been argued that the involvement of marginalized stakeholders, such as local communities, in multi-stakeholder initiatives is important it is not always achieved due to narrow inclusiveness, inequality in decision-making, power issues and conflicts. While more knowledge is being developed on tactics to address conflicts in multi-stakeholder initiatives, little is known about how challenges related to the involvement of marginalized stakeholders can be overcome during the course of multi-stakeholder initiatives to maintain inclusiveness and procedural fairness (Khazaei et al., 2015; Lu et al., 2018).

### The Circular Economy and Multi-stakeholder Initiatives

The circular economy is a key approach for sustainable development, offering a systematic solution to the waste of resources and environmental pollution caused by current consumption and production patterns (Chen et al., 2020). Following a review, Kirchherr et al. (2017) provide the following definition: ‘the circular economy is an economic system that replaces the ‘end-of-life’ concept with reducing, reusing, recycling and recovering materials in production, distribution and consumption processes and simultaneously generating environmental quality, economic prosperity and social equity to the benefit of current and future generations’. The literature proposes that multi-stakeholder initiatives are important in the context of the circular economy, as circularity requires the collaboration of several stakeholders and is too complex to be handled by one actor alone (Ghisellini et al., 2016). This is due to circular economy’s focus on value preservation, a collective value that can only be realized when actors collaborate to create various types of resource loops through recycling, conversion and the substitution of materials (Jonker & Faber, 2019). For example, in order for businesses to close resource loops, manufacturers need to adopt reusable materials and customers have to return products.

While research has emphasized the need for multi-stakeholder approaches in the circular economy, limited emphasis has been placed on the involvement of local communities in such an approach. The circular economy literature has been critiqued as being too narrow in its focus on environmental and economic objectives, and so failing to include societal participation and address societal perspectives (Millar et al., 2019; Murray et al., 2017). These aspects are important in order to transform consumption patterns and lifestyles and change the course of the current unsustainable economic paradigm (Millar et al., 2019). This is especially important in the context of cities and neighbourhoods where local communities can play a role by leading sustainable lifestyles, engaging in co-creating future visions and participating in governance (Fratini et al., 2019; Pomponi & Moncaster, 2017; Prendeville et al., 2018). However, also in this context project implementation is dominated by businesses and other large incumbent actors (Prendeville et al., 2018), which can lead to negative implications for local communities, for example, in the case of sharing initiatives and access models (delivering products as services) which can erode citizen autonomy (Fratini et al., 2019). Furthermore, not involving societal perspectives could lead to rebound effects (Zink & Geyer, 2017). Circular economy approaches may for instance not reduce resource usage when secondary goods are less desirable to users or when customers increase their consumption due to the lower prices provided by circular economy approaches (Zink & Geyer, 2017). Other critiques on the circular economy concept include its diffused limits, unclear theoretical grounds and problematic implementation which faces several structural obstacles (Corvellec et al., 2021).

In conclusion, it has been argued that adopting participatory and multi-stakeholder approaches, including local communities, is important in the circular economy context to enable legitimate decision-making processes and increase social and environmental benefits (Geissdoerfer et al., 2017; Inigo & Blok, 2019; Murray et al., 2017). However, it is still unclear how local communities can be involved in these initiatives, which are currently dominated by business actors. 

## Method

### Case Description

In order answer the research question—How can marginalized stakeholders, and local communities in particular, be successfully involved during the course of a multi-stakeholder initiative?—This study focussed on an initiative to involve local communities, next to other stakeholders, in the design and implementation of circular economy approaches in a low-income neighbourhood in the Netherlands. In this section, we firstly describe the neighbourhood, thereafter we elaborate on the initiative and its aims, and finally we highlight the different actors that were involved in the initiative.

The neighbourhood that was addressed in this study had around 4000 inhabitants with 80% of the houses owned by a local housing association. The neighbourhood had been classified as one of the poorest neighbourhoods in the region (17.4% of inhabitants had an income below the Dutch poverty line, while the national average is 5.2% of inhabitants) due to unemployment (21% of the labour force) and health challenges. Furthermore, there were a multitude of social challenges in the neighbourhood including nuisance, social isolation, waste and addictions. For example, 17% of the inhabitants reported nuisance due to neighbours and 17% of all individuals handled by the provincial social services department were individuals in the neighbourhood, while only 4% of the total number of inhabitants of the province were located in the neighbourhood. Simultaneously, most buildings in the neighbourhood had been built in the 1960s and were in need of large-scale renewal. Despite the challenges, the neighbourhood also possessed several strengths including multiple green spaces, a core of involved community members and an increasing amount of community initiatives.

Due to the challenges in the neighbourhood, the local housing association, in cooperation with the municipality, led and funded the development of an action plan for the neighbourhood. This plan involved an extensive neighbourhood renewal, in which 576 houses and 24 apartment buildings would be renovated or demolished and rebuild. Furthermore, multiple social challenges would be addressed in this process, for instance by increasing diversity in the types of housing and including more green and social spaces in the neighbourhood. An important role for the circular economy was highlighted in the action plan, for instance by reusing materials and implementing sharing principles. The ambition was set to transform the neighbourhood into one of the first circular neighbourhoods in the region. However, prior to the research, the plans were unclear, especially in terms of how circular economy approaches could be implemented in the neighbourhood. Therefore, as part of the action plan, an initiative involving multiple stakeholders was established with the aim to design and implement circular economy approaches in the neighbourhood in close cooperation with community members. We understand this initiative as a local partnership for problem solving and idea generation on a community level, similar to the Grainger Town Project discussed by Roloff (2008), because the initiative (1) addressed social and environmental issues in the neighbourhood, (2) involved diverse local stakeholders on a voluntary basis, (3) enabled local stakeholders, that were not representatives of organizations, to participate in decision-making and (4) stimulated dialogue among the involved stakeholders to enable collective action (Baumann-Pauly et al., 2017; Zimmermann et al., 2021). It is important to note that the initiative was terminated early due to the Covid-19 pandemic (further details can be found in the results section). While the initiative was executed and circular economy approaches were being designed in teams of diverse stakeholders, it was terminated before these approaches could be implemented in the neighbourhood.

**Fig. 2:**
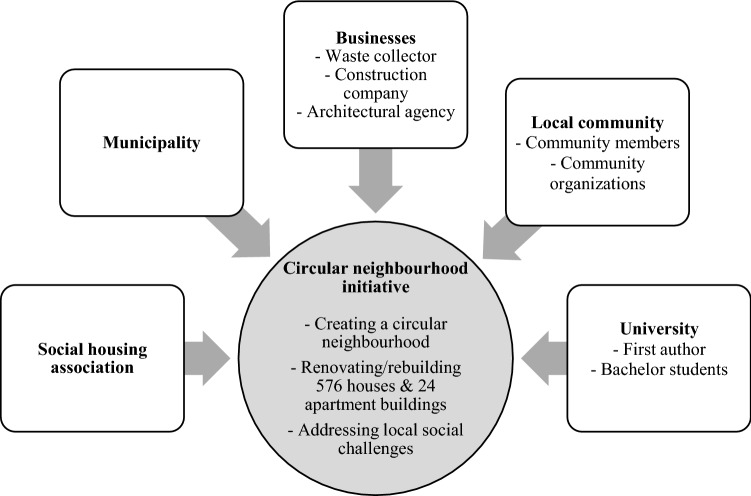
Stakeholders involved in the circular neighbourhood initiative

The initiative involved different stakeholders (see Fig. 2). First, the initiative was established, designed, executed and funded by a local housing association. Dutch social housing associations are private non-profit-making organizations with social goals providing low-income communities with affordable housing and improving their overall well-being. The housing association addressed in this paper rented out over 20,000 houses and had 185 employees. Relationships with communities, and tenants in particular, were important for the association and these stakeholders participated in new initiatives through information sessions and consultations. The housing association had been a national leader in the adoption of environmental approaches, for example by constructing energy neutral houses.

The local municipality was responsible for laying the foundations of the action plan for the neighbourhood in collaboration with the housing association. Furthermore, the municipality assisted the housing association in designing and executing the initiative. The municipality encompassed 123,000 inhabitants and was located in the North of the Netherlands. The circular economy had received increased attention in the municipality and a circular economy network organization, with over 100 members from business, government and civil society, and several circular economy projects with local businesses had been established in the years preceding the project.

Several businesses were involved in the initiative and its execution. First, a medium-sized (450 employees) local waste collector with high circular ambitions, including the aim to become the most circular collector and processor of household waste in the Netherlands. Second, a large (1950 employees) Dutch construction company, active in the local area. Third, a small (80 employees) design and architectural agency from the area. The construction company and architectural agency were collaborating on multiple novel circular innovations, including the design and construction of modular housing concepts. These businesses were seen as important for the implementation of circular economy approaches and technologies in the neighbourhood, such as modular housing techniques and new waste collection methods.

The local community, including individual community members (4000 inhabitants) and community organizations (such as a community centre, social working space and neighbourhood company), was involved in the initiative. The vulnerable position of community members was emphasized due to several challenges including unemployment, poverty, social isolation and addictions. Therefore, we view the local community as a whole as a marginalized stakeholder. Concerns were raised about the potential negative impacts of circular economy approaches on the community due to an increase in living expenses and a corresponding reduction in well-being. To prevent negative social consequences and increase support from community members, the importance of involving the community in the design and implementation of circular economy approaches in the neighbourhood was emphasized by the housing association. However, prior to the research, it was unclear how this could be achieved and it was emphasized that community involvement may be difficult due to the vulnerable position of community members and their limited awareness of the circular economy.

The local university was represented by the first author who joined the housing association with the objective to collaboratively design, execute and evaluate how the local community, next to other stakeholders, could be involved in the design and implementation of circular economy approaches in the neighbourhood. Furthermore, several bachelor students (BSc Global Responsibility and Leadership) of the university were involved in the execution of the initiative.

### Action Research Approach

An action research approach was adopted to answer our main research question: how can marginalized stakeholders, and local communities in particular, be successfully involved during the course of a multi-stakeholder initiative? In this section, we first elaborate on our action research methodology, thereafter we highlight the dual role of the researchers in this process, and lastly, we explain the action research cycle steps used in this research, elaborating on the data collection methods used during each step.

Action research has the dual purpose of advancing knowledge and contributing to the practical concerns of individuals by joint collaboration (Rapoport, 1970). This means that the researcher is embedded in an organization and contributes to generating the phenomena that are intended to be analysed (Perrot, 2017). In this way, data are not only obtained, but also generated through collaboration between the researcher and organizational members (Susman & Everd, 1978). Susman and Everd (1978) argue that rigour can be achieved in action research through an iterative process of data collection and analysis and the systematic triangulation of multiple perspectives and data sources. Action research fits our research purposes as (1) we are guided by a research topic that emerged from a real-world organization, (2) our research is intended to have real-world effects and involves real people in real settings and (3) our research requires a collaborative involvement with different organizations (Rapoport, 1970, p. 499).

The action research methodology required us to work as co-creators and co-learners with the stakeholders involved in the initiative. The action research collaboration started in September 2018 and terminated at the end of December 2020. For this collaboration, the first author joined the strategy department of the housing association, working dually at the housing association and university. For this arrangement the first author received a non-paid position at the housing association as an intern/researcher; employees were informed that the first author would observe the initiative and assist where possible using insights from the observations, interviews and literature. The second author was involved at a distance, focussing on reviewing and interpreting the data, without directly engaging in the initiative.

The role of the first author in the initiative was to assist in developing and executing the initiative, while leading its evaluation. The first author had more expert knowledge on the circular economy compared to most of the participants from working on these topics (in particular the circular economy in the build environment) for several years prior. Furthermore, the first author regarded the involvement of community members and social elements as important due to her prior knowledge. The first author thus influenced the initiative, by providing insights from the literature, following her own experience, to guide the initiative and reflecting on the activities with the participants. The dual role helped to bridge the gap between the theoretical and practical (Bartunek, 2007), but also introduced ethical concerns regarding the power of the researchers and the potential for the researchers to exploit participants for their own interests. To reduce these concerns, we made sure that all the participants knew and respected the first author’s combined role. This was achieved by introducing the researcher to all participants and asking for their approval and informed consent for the researchers’ involvement. To reduce the potential for patronization and exploitation by the researcher, the participants were included in and given the lead over the decision-making process in all phases of the action research process.

**Fig. 3:**
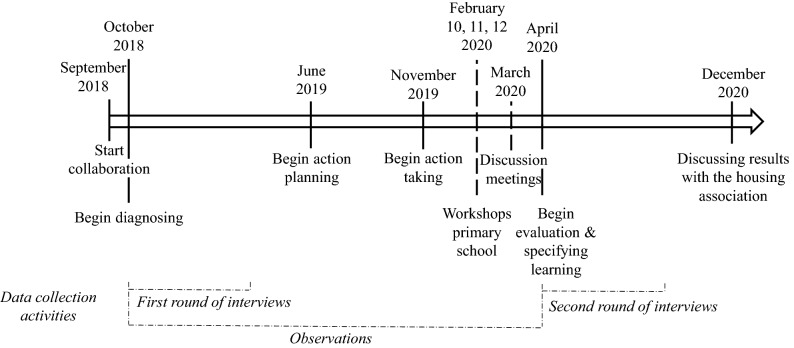
Timeline of the research activities

The action research cycle steps proposed by Susman and Everd (1978) were followed to conduct the research. The steps include (1) diagnosing: identifying and defining the situation, (2) action planning: collaboration between the researcher and practitioners to consider alternative remedies to a problem, (3) action taking: the implementation of the planned action, (4) evaluating: studying the consequences of the action and (5) specifying learning: identifying general findings. Although we followed these steps, our evaluation already started during the action planning step. Different data collection techniques were used during the action research cycle steps (Susman & Everd, 1978) including observations (see Table 1), interviews (see Table 2), and archival data (including documents on the neighbourhood, the action plan and the initiative, and internal/external communications such as e-mails and messages on the housing association’s intranet). The next sections will explore the action research cycle steps in detail, elaborating on the data collection methods and role of the researcher in each step. Figure 3 provides a timeline of the research activities.

**Table 1:** Observations during the diagnosing, action planning, action taking and evaluation steps

Occasion	Number of times	Duration	Total
Observations in office	1 time per week, for 40 weeks	8 h a day	320 h
Site visits to the neighbourhood	2	2–3 h per visit	5 h
Action planning meetings	10	1–2 h per meeting	15 h
Workshops at the school	2	4 h per workshop	8 h
Discussion meetings	4	2 h per meeting	8 h
			356 h

**Table 2:** Interviewees diagnosing step

Stakeholder	Interviewee function	Duration (in minutes)
Housing association	1. Rental collections	30
	2. Rental collections	35
	3. Portfolio analyst	30
	4. Advisor housing	40
	5. Tenant affairs	35
	6. Executive secretary	40
	7. Maintenance advisor	30
	8. Project leader social affairs	50
	9. Construction professional	40
	10. Rental manager	40
Social team	11. District manager	40
Social working space	12. Supervisor & manager	50
Community centre	13. Manager	30
	14. Project manager	25
Tenant association	15. Board member	30
Community space & restaurant	16. Owner	40
Second-hand shop	17. Manager	45
School	18. Sustainability coordinator	45
Municipality	19. Project manager	50
	20. Senior policy officer	60
	21. Sustainability officer	60
Waste processor	22. Director	50
Builder/architect	23. Manager circularity	60

#### Diagnosing

The diagnosing step started in October 2018 after initial discussions at the housing association between the researchers, the manager of the strategy department, the strategic relations manager and asset manager. Within these meetings it was confirmed that the idea was to collaboratively develop, execute and evaluate an initiative for involving the community, next to other stakeholders, in the design and implementation of circular economy approaches in the neighbourhood. In order to build a solid research base, we firstly focussed on developing an increased understanding of the context, including the neighbourhood and its challenges, and the different involved stakeholders, as well as the initial understandings of the involved stakeholders of circular economy approaches in the neighbourhood and the potential for community involvement. Data collection during this phase involved two visits to the neighbourhood, 23 semi-structured interviews (Table 2), and the analysis of archival data, including documents on the neighbourhood and the action plan. Interviewees were identified based on their involvement in the action plan and initiative. Furthermore, interviewees from the community were identified by asking the housing association which individuals or organizations had an important function in the community. We focussed on interviewing different community members and organizations that played a pro-active role in the community including shops, the community centre and social team. We decided to focus on these groups in this phase in order to develop a general understanding of the neighbourhood. The duration of the interviews was between 30 and 60 min and all interviews were recorded and transcribed. Interviewees were asked about their role in developing the action plan for the neighbourhood, their perspectives on the challenges and strengths of the neighbourhood, their understandings of the circular economy and the adoption of circular economy approaches in the neighbourhood, and their perspective on involving the community in the design and implementation of circular economy approaches in the neighbourhood (interview guide is provided in “Appendix A”). During this step the role of the researcher was to build a solid research base and identify the possibilities and challenges for the involvement of the community, next to other stakeholders, in the design and implementation of circular economy approaches in the neighbourhood.

#### Action Planning

After analysing the materials from the diagnosing step, the action planning step started in June 2019. The aim of this step was to design an initiative for involving the community, next to other stakeholders, in the design and implementation of circular economy approaches in the neighbourhood. Action planning was conducted in cooperation with a team of housing association employees who had responsibility for the neighbourhood and/or the action plan. These included (1) the manager of the strategy department, (2) the strategic relations manager, (3) the asset manager, (4) the tenant affairs advisor and (5) the social affairs project leader. In total 10 meetings were held (where at least two of the above employees joined) in which the findings of the diagnosing step and insights from the multi-stakeholder initiative literature were discussed. Furthermore, the meetings involved brainstorming activities to design ways in which the community could be involved. The researcher took ethnographic notes from the meetings, which were transcribed. The role of the researcher during this step was to share the findings from the diagnosing step and insights from the literature and join the brainstorming activities.

#### Action Taking

During the action taking step the designed initiative was executed (more information on the initiative can be found in the results section). The initiative involved organizing two workshops at a local primary school and multiple discussion meetings with different stakeholders (Table 3). The workshops at the primary school were planned during November 2019 and executed in February 2020. Thereafter, multiple discussion meetings were planned and executed in March 2020. The researcher joined all workshops and meetings and took notes. The researcher had three roles in the action taking step including (1) jointly planning the workshops and discussion meetings, (2) assisting in giving the workshops at the primary school, by providing two short presentations for the school in particular and (3) managing relationships with the involved stakeholders, including making phone calls and sending e-mails to the invited stakeholders.

**Table 3:** Stakeholders involved in action taking

Activity	Stakeholder	Employee/actor	Role
Workshops at the primary school	Housing association	Tenant affairs advisor	Planning & assisting in the workshops
		Project leader social affairs	
	Primary school	Teachers	Authority in the class
		Director	Planning the workshops
		22 students (7th & 8th grade)	Participating in the workshops, making documentaries
	Circular network organization	Educational manager	Giving the workshops
	University	Researcher	Planning & giving the workshops & coordinating stakeholders
		Bachelor students (14)	Assisting in the workshops
	Community organizations	Neighbourhood company, energy manager, social working space, community centre, social team, concierge, sport clubs	Participating in the workshops (being interview for the documentaries)
	Community	Community members in the neighbourhood	
		Parents of the students	Attending the premiere of the documentaries
Discussion meetings	Housing association	Tenant affairs advisor	Planning & coordinating & attending
		Project leader social affairs	
		Strategic relations manager	
		Director	Attending
	Municipality	District manager	Planning & attending
		Sustainability manager	Attending
	Primary school	Director	Planning & chairing & attending
		22 students	Showing the documentaries
	University	Researcher	Planning & attending & coordinating stakeholders
	Circular network organization	Educational manager	Planning & attending
		Director	Attending
	Builder/architect	Strategy manager	
		Architect	
	Waste processor	Director	
	Community	Interested community members	
	Community organizations	Neighbourhood company, energy manager, social working space, community centre, social team, concierge, sport clubs	

#### Evaluating

After the action taking step, the evaluation step started in April 2020 in which the initiative was evaluated. This was done through interviews with multiple stakeholders after the discussion meetings took place, as well as through observations and the analysis of archival data concerning the initiative and action plan for the neighbourhood (including documents on the neighbourhood, the action plan and the initiative, and internal/external communications such as e-mails and messages on the housing association’s intranet). We asked stakeholders to evaluate the discussion meetings, including questions like ‘How did you experience working together with the other stakeholders during the meetings?’ and ‘Do you think the involvement of community members in the discussion meetings was valuable for the design and implementation of circular economy approaches in the neighbourhood?’ (Interview guide is provided in “Appendix B”). Evaluation was not solely conducted during the evaluation step, as during each step evaluations were collected through informal conversations with the involved stakeholders during or right after the activities took place. The role of the researcher in this step was to evaluate the initiative and encourage the involved stakeholders to reflect on the initiative.

#### Specifying Learning

All materials, including the interviews, observations and archival data, were coded using a 1st- and 2nd-order coding methodology (Gioia et al., 2012) in Atlas.ti 8 (data structure is provided as supplementary material). Data analysis involved two rounds: one round focussing on data gathered during the diagnosing step and one round performed after the evaluation step. In the two analysis rounds, a similar data analysis approach was adopted which involved three stages. First, we conducted text queries to search for keywords and phrases, as informed by our research question. For example, when reading the transcripts, we searched for mentions of challenges experienced in the involvement of the local community, labelling them as such at this first stage. Different data sources, including interviews, observations and archival data, were used to validate the researchers’ interpretations. After re-reading the interviews and other data sources, we gradually combined the original labels into first-order codes. Second, we combined the first-order codes into second-order themes, to create a coherent storyline that articulated our understanding of the approaches for, and challenges encountered during, the involvement of the community in the multi-stakeholder initiative. Finally, we gathered the second-order themes into aggregate dimensions. The two researchers coded the data working independently and a coding comparison, were the researchers met several times and discussed the emerging codes, was performed to increase reliability between the researchers. During these meetings no major differences between the coding of the two researchers were found, minor adjustments were made to the labelling of the codes. Our data analysis also involved discussing the emerging themes and dimensions with the participants for validation purposes. We did this during the action planning step, discussing and agreeing on themes emerging from the data gathered during the diagnosing step, and in an evaluation meeting in December 2020, discussing and agreeing on themes and dimensions emerging from all data.

In the coding process we built on the previously discussed multi-stakeholder initiative literature, we analysed our data against the literature, eliminating concepts and theories that did not match the emerging patterns. This corresponds to an abductive approach, which focusses on the continuous interplay between theory and empirical observations with the aim of integrating these streams, as well as advancing knowledge, through an in‐depth analysis (Dubois & Gadde, 2002). Abduction is useful to explain new and surprising empirical data through the elaboration, modification, or combination of pre-existing concepts as it confronts theory with the empirical world (Richardson & Kramer, 2006). The abductive approach is thus useful when the objective is to discover new things, other variables or relationships, leading to the generation of new concepts and the development of theory, rather than confirming existing theory (Dubois & Gadde, 2002).

## Results

This section will specify the results of our study. First, we detail our findings regarding the initial obstacles that were experienced regarding the involvement of the local community, as a marginalized stakeholder group, in the initiative. Second, we will explain how the planned initiative was designed and executed. Third, we will focus on the challenges that were experienced during the initiative. Lastly, we will elaborate on the outcomes of the initiative.

### Initial Obstacles Regarding the Involvement of Community Members

During the diagnosing step several initial obstacles to involving the community, next to other stakeholders, in the design and implementation of circular economy approaches in the neighbourhood were identified, including the need to (1) deal with different understandings of the goals of circular economy approaches and (2) overcome the difficulty of involving the local community, due to either reluctance from the other stakeholders or from community members themselves.

#### Different Stakeholder Understandings

Several different understandings of the goals circular economy approaches could serve in the neighbourhood were indicated by the involved stakeholders, including an environmental, economic and social understanding (see Table 4).

**Table 4:** Initial understandings of the goals of circular economy approaches (based on the diagnosing step findings)

Category	Category explanation	Example quotes	Stakeholders that mentioned the category
Environmental understanding	Interviewee emphasizes the achievement of environmental goals through the adoption of circular economy approaches such as reducing waste and material use, achieved through the adoption of new building and recycling techniques	Circularity is about reducing waste, through recycling and improving the quality of the environment, because there is a lot of waste. (Interview: waste processor, director) I really relate the circular economy to reducing our impact on the environment. (Interview: housing association, executive secretary) The circular economy is about the process of making something, in our case houses, making sure that we do not throw materials away but recycle them in order to decrease our impacts on the environment. We need smart solutions for that, we need new technologies. (Interview: housing association, advisor housing)	Builder Architect Waste processor Housing association: construction professional, advisor housing, executive secretary Municipality: sustainability officer
Economic understanding	Interviewee emphasizes the achievement of economic goals through the adoption of circular economy approaches including efficiency and cost reductions	I think it is about efficiency, using your materials in an efficient way and reducing your costs. (Interview: housing association, portfolio analyst) It is about efficiency and reducing costs, you have to evaluate carefully if reusing water or warmth leads to decreased costs in the neighbourhood. Otherwise, circular economy approaches might not be worthwhile. (Interview: municipality, senior policy officer)	Municipality: senior policy officer, project manager Housing association: portfolio analyst Social team
Social understanding	Interviewee emphasizes the achievement of social goals through the adoption of circular economy approaches including revaluing talents and improving well-being	With circularity we can reconnect to the community, revaluing and using their qualities, talents and positions in society. (Proposal initiative, housing association) We really focus in our shop on how we can reuse clothing and give people a second chance. (Interview: second-hand shop, manager) I see the circular economy as a social practice, revaluing the talents of people in the neighbourhood. We have to find out what people want and can do and make use of these talents, instead of leaving people without a job at home. (Interview: community space, owner)	Community space Social team Second-hand shop Housing association: social affairs project leader
Fluid understanding	Interviewee emphasizes the potential to achieve multiple goals (social, environmental and/or economic) through the adoption of circular economy approaches	We do often start with the ecological goals, however, I also think social and financial benefits can be achieved with circularity. (Interview: builder, manager circularity) I connect circularity to a better environment. At first, I never thought about the social aspects, like the happiness of residents. But now I see that that may also be an interesting connection. (Interview: housing association, executive secretary)	Builder Housing association: executive secretary Social team
Fixed understanding	Interviewee emphasizes that the adoption of circular economy approaches may only lead to the achievement of certain goals, excluding other goals	We focus on the achievement of environmental goals, through technical solutions, not really looking at social aspects (Interview: municipality, sustainability officer) I think the circular economy is about buildings, adopting new technologies in houses, I do not see a link between the circular economy and the social problems in the neighbourhood. (Interview: housing association, construction professional)	Housing association: construction professional Municipality: sustainability officer, project manager

First, interviewees working on the construction of houses (including the builder, architect and housing association’s construction professional and advisor housing), understood the goals of circular economy approaches as being environmental in nature. Within this understanding the importance of reusing and recycling technologies in the neighbourhood to achieve environmental goals was emphasized. Second, several interviewees, mainly those supervising and supporting construction projects (including the municipality and the housing association’s portfolio analyst), had a more economic understanding of the goals of circular economy approaches, where the focus was on implementing circular economy approaches to reduce costs. Finally, a third understanding of the goals circular economy approaches was indicated by interviewees in the community and those with close relationships to the community (including the community space, social team and the housing association’s social affairs project leader), which was more socially oriented. These interviewees understood the circular economy as a way to improve the well-being of community members through revaluing their qualities.

Multiple interviewees were ‘fluid’ in their understanding of the goals of circular economy approaches, highlighting technical, environmental, economic and social benefits. However, most interviewees only emphasized one or two aspects and some did not see a relation with other aspects at all. Multiple understandings of the goals of circular economy approaches also existed within organizations, especially in the housing association. These differences were mostly related to the functions of the interviewees. For instance, individuals working in the construction of buildings mostly emphasized environmental goals, whereas individuals working closely with or in the community emphasized social goals. The resulting challenge was to find ways to deal with and combine these different understandings during the initiative.

#### Difficulties with Involving the Local Community

There were difficulties with regards to involving the local community as there was a reluctance among multiple stakeholders to involve the local community in the design and implementation of circular economy approaches due to several reasons (see Table 5).

**Table 5:** Initial perspectives on community involvement (based on the diagnosing step findings)

Category	Category explanation	Example quotes	Stakeholders that mentioned the category
Community involvement is not needed	Perception that it may not be needed to involve the community in the initiative as circular approaches can be adopted without community members noticing it	We have to unburden the community, if we want to have the ultimate circular economy, we have to adopt it in such a way that community members will not notice it. (Interview: municipality, senior policy officer) We expected that community members may not like it if they can see that their house is circular. So, we decided to make sure they wouldn’t. In that case you don’t really need much from them. (Interview: builder, manager circularity) We can adopt circular building practices in the neighbourhood, making it easier to take the buildings apart. We developed these technologies in such a way that people living in the houses will not notice it, therefore there is no need to actively involve them. (Interview: builder, manager circularity)	Municipality: senior policy officer Builder Housing association: construction professional, maintenance advisor
Community involvement is difficult	Perception that community involvement in the initiative is difficult to achieve, mainly because it is time-intense, involves high costs and a lack of understanding among community members	With these kinds of things, you are really talking about intensive processes with many meetings. Then you do really have think about it, if it is realistic to do. (Interview: waste processor, director) In order to involve local people in circular economy approaches we need new methods in which we keep intensive contact with them. The question is if we have the time and money for such a process. (Interview: municipality, sustainability officer) I am afraid that when you talk about circular economy approaches, many community members will not understand what you are talking about. You have to find a way to talk about these topics with community members, making it relevant for them. (Interview: social team, district manager)	Housing association: portfolio analyst, executive secretary Municipality: sustainability officer, project manager Waste processor Builder Social team
Community involvement may lead to negative impacts	Perception that community involvement in the initiative may lead to negative impacts such as promises that cannot be made or a burden on communities	Maybe it is better to not involve the community. In another project we did involve the community in the beginning, however, afterwards it was decided that no investments would be made in the project. That led to disappointment. (Interview: housing association, advisor housing) We really have to think about if community members want this. Because maybe we will only burden them with requirements for circularity. (Interview: housing association, project leader social affairs)	Housing association: portfolio analyst, executive secretary, rental collectors, project leader social affairs, advisor housing Builder Municipality: sustainability officer
Community involvement may not have any impact	Perception that community involvement may not have an impact in the initiative, as the input of community members may not be taken into account/ taken seriously	Well, I have to say that I am reluctant, I don’t directly feel like joining because last time we did, it did not have much impact. (Interview: tenant association, board member) There is an increasing feeling among community members that, if they put efforts in collaborating on topics, especially on less tangible topics such as the circular economy, nothing will be done with their efforts. Therefore, the motivation to become involved is low. (Interview: social team, district manager)	Social team Tenant association Community centre Housing association: project leader social affairs

First, most stakeholders working on the construction of houses (including the builder and the housing association’s construction professional and maintenance advisor), felt that there was no need to involve community members as they would likely not be interested in nor affected by circular economy approaches. Second, stakeholders supervising and supporting construction projects (including the municipality and members of the housing association) did recognize the importance of involving the community in order to increase their support for circular economy approaches and create social benefits. However, these stakeholders, especially members of the municipality, also stressed that involving the community would be a complex and costly process. Additionally, multiple stakeholders with close contact to the community (including the housing association’s project leader social affairs and social team) mentioned that it can be difficult for community members to get involved in circular economy approaches as they may not know what it entails in practice. Third, several members of the housing association supervising construction projects (including the portfolio analyst and executive secretary) and those working in close contact with the community (including the rental collectors and project leader social affairs) stressed that community involvement might lead to expectations that could not be met and pose a burden on vulnerable community members. Finally, it was stressed by stakeholders in the community (including the community centre) and by those with close contact to the community (including the social team) that the community may be reluctant to become involved in circular economy approaches. For instance, some interviewees feared that, when community members would become involved in circular economy approaches, nothing would be done with their efforts. 

To conclude, the interviews revealed that stakeholders working on the construction of houses and those managing/supporting construction projects (including members of the housing association, municipality and builder/architect) found it difficult to involve the community in the initiative, while stakeholders in the community and those with close contact to the community (including the social team, community space and members of the housing association) stressed the challenges of involving communities in the initiative and the potential reluctance of community members to become involved.

### The Initiative

**Fig. 4:**
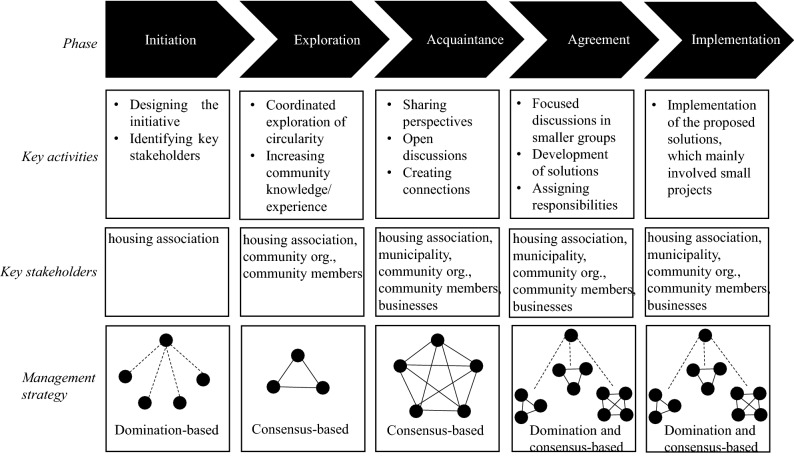
The phases of the initiative

Following the insights from the diagnosing step and multi-stakeholder initiative literature, an initiative to involve the community in the design and implementation of circular economy approaches was designed and executed in the action planning and taking steps. This initiative included several phases (Fig. 4).

The initiative was started by the housing association, involving the design of the initiative and the identification of key stakeholders. An additional phase (not included in issue-focussed stakeholder management) was deemed necessary in order to enable the community to become familiar with the circular economy. This was done in the exploration phase, which had several aims: (1) increasing the familiarity of community members with the circular economy, (2) making the connection between the circular economy and the community more tangible for other stakeholders and (3) creating enthusiasm for the circular economy in the neighbourhood. The exploration phase involved a coordinated exploration of what the circular economy could mean in the neighbourhood, highlighting social, economic and environmental aspects. In order to do this, the housing association collaborated with primary school students who made short documentaries about the different aspects of the circular economy in their neighbourhood. It was decided to collaborate with a local primary school due to the central position of the school in the neighbourhood, its ability to link community members and its capacity to create an enthusiastic and open atmosphere. For their documentaries, primary school students interviewed community members and organizations in the neighbourhood regarding their understanding of the circular economy and ongoing circular economy projects, such as a local second-hand clothing store (see Online Appendix C for an example).

The acquaintance phase involved discussion meetings in which the different stakeholders met each other, learned about each other’s perspectives and exchanged opinions. Within this phase the documentaries made by the students served as a conversation starter, enabling open discussions among stakeholders. While the discussions were organized around the documentaries, the aim was to keep them open in terms of the topics that could be discussed. After the acquaintance phase, the agreement phase started in which stakeholders discussed their perspectives to arrive at common approaches. In this phase, stakeholder teams were formed around different topics including waste, reuse and repair, revaluing talents, and sharing. For instance, for the topic ‘waste’ the team involved several community members, the community centre, the municipality and the waste processor. These teams had focussed discussions on the topics and thought about circular economy approaches that could be implemented in the neighbourhood. Furthermore, the teams would assign responsibilities among its members for the implementation of the approaches. The housing association served as a coordinator of the teams and activities during the agreement phase and each team was joined by at least one housing association employee. The designed approaches would be implemented during the implementation phase; however, this was not realized during the course of this research due to the COVID-19 pandemic.

### Challenges Encountered During the Planning and Execution of the Initiative

During the evaluation and specifying learning steps it was identified that three main challenges were encountered during the planning and execution of the initiative, including (1) ensuring equality, (2) dealing with disagreement and (3) reducing uncertainty (see Table 6). We will discuss these challenges in turn and illustrate how they were managed during the initiative.

**Table 6:** Main challenges experienced during the planning and execution of the initiative

Category	Category explanation	Example quotes	Example observations
Inequality	Inequality was experienced during the initiative in terms of the perspectives of some stakeholders getting more attention compared to other stakeholders. This related mostly to the perspectives of the builder, waste processor, housing association and municipality getting more attention compared to the perspectives of community members. Furthermore, the perspectives of some community members got more attention than those of others	Businesses are more used to work with other businesses and stakeholders such as the municipality. Therefore, these actors find each other more easily in the discussions and may forget to include the perspectives of the community. (Evaluation after the discussion meetings: community centre, manager) We see that some partners are less open to the suggestions of community members. They are also scared that community members will ask something that they cannot provide. (Evaluation after discussion meetings: housing association, strategic relations manager) I was very excited about this project; I already told my two interns to do some research about the circular economy. I really hoped they would involve the whole community this time. (Phone call planning workshops school: neighbourhood company, manager)	During the discussion the representatives from the municipality and housing association are more talkative compared to the community members at the table. Most of them only talk when a direct question is asked to them, which does not often happen. (Researcher notes, discussion meetings) The representative of the builder is listing a lot of concerns for the proposed project (the reuse of materials by community members), already dropping the idea before the community members are able to voice their opinion. (Researcher notes, discussion meetings)
Disagreement about stakeholder inclusion	Disagreement, among those involved in the planning of the initiative, about which community stakeholders to include in the initiative was experienced	I think we should invite the neighbourhood company and second-hand store. They really do something with it. I don’t think the other community members are our target group in this project. I don’t agree with that, circularity can be about more than just these things; it can mean something for the other community members as well (Planning discussion meetings: housing association tenant affairs advisor & social affairs project leader) The first discussion meetings were quite difficult. Afterwards, she [the tenant affairs advisor] asked me if I was still happy about my decision to include all these community members. But yes, I really think it was a good idea (Evaluation after discussion meetings; housing association, social affairs project leader)	The tenant affairs advisor looks unhappy with the decision to include more community members in the project. However, the social affairs project leader and municipality district manager are in agreement and quickly move the conversation to another subject. (Researcher notes, planning discussion meetings)
Disagreement about the goals of circular economy approaches	Disagreement about the goals on which the circular economy approaches would focus on was experienced among the different actors involved in the discussion meetings	Especially the meetings with the building companies were difficult, as there were people from the social domain that had radically different ideas about the circular economy. We had to find ways to combine these, which involved a lot of time and frustration (Evaluation after the discussion meetings: housing association, strategy manager) It sometimes felt like a vicious cycle where we did not connect with each other and kept talking past each other. (Evaluation after the discussion meetings: circular network organization, educational manager)	The builder tries to steer the conversation in the direction of the reuse of materials in new houses for environmental gains, while the involved community members are more interested in if they can share materials in order to save money, mostly for use in their sheds, and if this would be allowed. It seems like two different conversations are going on. (Researcher notes, discussion meetings) The community members at the table seem unhappy with the focus on waste, and how to best collect it, in the discussion. Therefore, they get more silent during the conversation. I see more enthusiasm from them when the conversation moves to their ability to share products during a picnic, thinking about how they can create more social interaction in the neighbourhood. However, I observe the exact opposite pattern for the waste manager, who seems to get frustrated when the topic moves away from the environmental aspects (Researcher notes, discussion meetings)
Uncertainty regarding the outcomes of the initiative	The involved stakeholders experienced uncertainty about the potential outcomes of the initiative, which could result in their unwillingness to become involved in the initiative	I am unsure about this project, why is my opinion needed when in the end new rules for our waste will be set up without thinking about the people living here? (Workshop school: conversation between children & community member) I don’t know if this initiative is interesting for us. How are the results going to help our work here? (Planning discussion meetings: conversation between researcher and builder, strategy manager)	There have been three phone calls with the builder and architect in which they were reluctant to join the initiative. They seem afraid the results of this project will not be in their benefit. It is difficult to convince them of the benefits of this project and I am not sure how the project will proceed (Researcher notes, planning discussion meetings)
Uncertainty regarding stakeholder roles	The involved stakeholders experienced uncertainty about their roles in the initiative	I am not sure if we can add something to this initiative as I am not certain what our role should be as a partner in the construction value chain. What is the focus of circular economy approaches in this project and what are our responsibilities? (Planning discussion meetings: conversation between researcher and builder, strategy manager) I am not sure what to do at this moment. I like the ideas coming up, but I also think these things may not be related to our role as waste processor. (Evaluation after discussion meetings: waste processor, director)	The community members at the table want to talk more about their ability to share products. However, the waste manager does not seem to know how to deal with this request as he tells the community members that this is not his expertise and aims to steer the conversation in another direction. (Researcher notes, discussion meetings)

#### Ensuring Equality

Ensuring equality, especially between community members and the other involved stakeholders, became an important challenge during the initiative. Most of the involved stakeholders, such as businesses and the municipality, possessed more knowledge and resources to propose and execute circular economy approaches compared to community members. This could result in inequality, where the perspectives of the other stakeholders may receive more attention compared to the perspectives of community members. It was for instance argued that community members may feel emotionally overwhelmed due to the technical terms used and the diversity of actors and perspectives included and may therefore not be able or willing to share their perspectives:

We need to carefully plan the interactions among the involved stakeholders and the community. There are so many different opinions and assumptions involved, that community members might get overwhelmed or their opinions overshadowed. (Meeting planning discussion meetings: housing association—project leader social affairs)

Furthermore, not all community stakeholders and perspectives could be involved due to time and other project constraints. For example, some community organizations could not be involved in the exploration phase as the primary school students did not have time to interview all of them. This caused disappointment and, while they were invited for the acquaintance phase, some community organizations decided not to join after the initial disappointment.

The housing association decided to adopt domination-based strategies to ensure equality during the initiative. In these strategies, the housing association (sometimes in cooperation with the municipality) set the agenda, decided which stakeholders to include and steered interactions. For example, the housing association coordinated teams during the agreement and implementation phases and made sure approaches were feasible and beneficial for all stakeholders. The use of domination-based strategies to manage the initiative enabled the housing association to steer the relationships and outcomes in a positive direction for the community. However, it was also emphasized that domination-based strategies should be combined with consensus-based strategies. A consensus-based strategy was for example adopted in the acquaintance phase, where the involved stakeholders collectively set the agenda and openly shared their perspectives in relation to the documentaries made by the students. It was acknowledged by the housing association that the adoption of consensus-based strategies was important to allow for the emergence of new interactions and solutions:

We cannot fully plan the interactions upfront, it really depends on the individuals that are involved and their interactions. We need to give participants freedom to find new ways of working together. (Meeting planning discussion meetings: housing association—strategic relations manager)

Finding the right balance between domination-based and consensus-based strategies was difficult and required a long process of discussions among the housing association employees. For example, employees with a close relationship to the community applauded the adoption of a consensus-based strategy in the first phases of the initiative, as it enabled community members to share their perspectives and indicate important topics for them. However, employees working on the construction of houses argued that this was an unnecessary step which would delay the initiative. Instead, they argued that the housing association and municipality should decide on the topics to better guide the discussions. The employees agreed to go for the middle-ground, where community members and other stakeholders would be able to share their perspectives in the acquaintance phase and have more focussed discussions on topics specified by the housing association (based on the discussion in the acquaintance phase) in the agreement phase. However, the discussion was re-opened during the acquaintance and agreement phases, as some employees and stakeholders felt that the discussions needed more guidance.

#### Dealing with Disagreement

During the initiative, disagreement about two main issues arose. First, there was disagreement among the housing association employees regarding which stakeholders and perspectives to include in the initiative. This was mainly experienced during the initiation phase, as there were different opinions about which stakeholders and perspectives were relevant. Not all employees were in favour of including community members and the school, arguing that only inviting representatives of the community (such as the tenant association) and those directly working with the circular economy (such as the second-hand shop) would be sufficient. However, the housing association’s social affairs project leader convinced the other employees by stressing the challenges with community involvement in past projects relating to the energy transition (which frequently led to unsatisfied community members, as they felt they were unable to share their opinions and concerns in these projects).

Second, during the acquaintance and agreement phases there was a substantial level of disagreement about the goals on which the circular economy approaches should focus in the neighbourhood. While most stakeholder perspectives were not contradictory, combining them was difficult and required an extensive amount of time. Disagreement continued in the agreement phase, where some stakeholder teams ‘agreed to disagree’ and stopped looking for a shared perspective on the goals of the circular economy. Instead, they sought for agreement in a circular economy approach, in which the circular economy was treated as a common way of working through which diverse goals in the neighbourhood, involving economic, ecological and/or social goals, could be addressed:

I think in the end it did not really matter what we all thought about the circular economy. I think it was more important that we focused on finding ways to use circular economy approaches to work towards goals we all believe in and create a pleasant neighbourhood. (Evaluation after the discussion meetings: social working space—supervisor & manager)

For example, one team came up with the idea to organize a local marketplace where community members and community-based organizations could share and repair left-over or broken products and materials. This marketplace was a shared approach to address different goals, including increased social interactions (emphasized by the community and municipality), easy access to repairing facilities (emphasized by the community), reduced waste (emphasized by the municipality and waste processor) and the ability to raise awareness (emphasized by the waste processor).

While most teams did agree on a common approach, not all stakeholders felt ownership over this approach. For example, the builder involved in the reuse and repair team did agree with the common approach, enabling community members to use left-over materials for small at home projects (such as sheds), but did not feel like it had much to do with its own activities:

These projects are of course very nice for the community members; however, they do not have much relation to our activities. In this neighbourhood our focus should be on reusing materials in buildings. That will in the end result in more environmental gains. Providing some materials to community members is a side project that should be led by those in the community. (Evaluation after the discussion meetings: builder—strategy manager)

#### Reducing Uncertainty

Some stakeholders were unwilling to collaborate and share their perspectives during the initiative. This was mainly experienced among community members during the initiation and exploration phases. This unwillingness resulted from a lack of trust in and uncertainty about the initiative. The exploration phase assisted in reducing uncertainty among community members as the resulting documentaries highlighted different aspects of the circular economy in the neighbourhood in a language understandable for community members. However, uncertainty was still an important challenge during the initiative. Several stakeholders were for instance unwilling to collaborate in the acquaintance, agreement and implementation phases due to uncertainty regarding the circular economy approaches that would be developed and their potential contribution towards them. This argument was mostly used by stakeholders concerned with the construction of houses (including the builder and architect) who also felt that it was not necessary to actively engage community members in the formulation of circular economy approaches. The housing association and municipality tried to persuade these stakeholders to join by framing the initiative as an experiment for community involvement.

While uncertainty about the initiative and its outcomes could lead to a reluctance to collaborate, it also had a beneficial side. There was for example uncertainty about the way in which circular economy approaches would be implemented in the neighbourhood during the early phases of the initiative. This provided the space necessary to enable the open exploration of community perspectives in the exploration phase and the integration of these perspectives during later phases of the initiative:

I was surprised by what was already happening in the neighbourhood. You wouldn’t expect that the community adopts circular economy approaches, however in their way they do a lot already. If we would have imposed our own vision, we might not have gotten a look into how people in the neighbourhood are already thinking about waste and reuse. (Informal evaluation after the primary school workshops: housing association—advisor housing)

### Outcomes of the Initiative

During the evaluation step it was identified that the initiative resulted in several outcomes. First, new connections were established between some of the involved stakeholders with the intention to work together on shared goals such as assisting community members in finding suitable job opportunities. Second, the initiative generated enthusiasm for the circular economy in the neighbourhood. For example, after the exploration phase several community-based organizations expressed their interests in learning more about the circular economy to the researcher and housing association. Additionally, the involved stakeholders got the opportunity to experience the way in which circular economy approaches were already used in the neighbourhood, which was argued to be important for initiating new collaborations:

Parties, especially technically oriented parties such as builders, really had to visit the neighbourhood to see what happens there. This helped them to recognize the value of working together with community members on, for them, technically oriented topics such as the circular economy. (Evaluation after discussion meetings: housing association—strategic relations manager).

Furthermore, the ways in which circular economy approaches could be implemented in the neighbourhood became clearer:

The meetings with community members have led to new insights into the circular economy and the documentaries of the students are great. These things are definitely going to help us and the municipality to design a good action plan for the neighbourhood. (Intranet post: housing association—strategy manager)

However, the initiative did not lead to the direct implementation of circular economy approaches in the neighbourhood during the course of this research. Multiple potential ideas were mentioned by the stakeholder groups, such as placing new ‘creative’ waste bins in the neighbourhood, organizing a picnic for sharing unused products, and arranging a local marketplace. However, these projects were not implemented due to the early termination of the initiative. Due to the COVID‑19 pandemic multiple discussion meetings could not take place, as it was difficult to organize discussion meetings due to restrictions in terms of the number of individuals that could attend meetings. The housing association tried to get around these restrictions considering, for example, the adoption of online meetings or the organization of a picnic. However, these alternatives were difficult to execute (as not all community members had access to computers and the temperature was deemed too low for a comfortable picnic) and the housing association did not want to strain community members during the difficult time of the pandemic. After the restrictions were relaxed, the priorities of most stakeholders had shifted, and instead of focussing on conducting circular economy approaches with the community members, the reconstruction of the neighbourhood, which had experienced delays due to the pandemic, was given priority. While the housing association attempted to continue the initiative, the influence of the pandemic increased the difficulties of involving the community and increased time pressures among the other involved stakeholders (including the builder, architect and municipality), leading to its abandonment. While there were no direct effects of the initiative, in terms of the implementation of circular economy approaches, it was emphasized that in the long-term effects may be experienced:

The project and meetings planted a seed, not only in our organization, but also in the other involved stakeholders and in the neighbourhood itself. We will definitely think about this initiative again in the development of the action plan, investigating how we can integrate the circular economy and work with community members in new ways (Evaluation after discussion meetings: housing association—strategic relations manager)

## Discussion

The objective of this study was to investigate how marginalized stakeholders, and local communities in particular, could be successfully involved in a multi-stakeholder initiative. We contribute to knowledge regarding multi-stakeholder initiatives by revealing that the inclusion of marginalized stakeholders does not only require attention at the start of multi-stakeholder initiatives to eliminate challenges—as previously emphasized—but demands continuous management, and the balancing of three factors including uncertainty–certainty, disagreement–agreement, and domination- and consensus-based management. We outline each of these factors below, highlighting the importance of continuously managing these factors during the course of the initiative. Finally, we elaborate on the contributions of this study in the conclusion.

### Uncertainty

Our results indicated that during the initiative included stakeholders experienced a high level of uncertainty. We understand uncertainty here as the stakeholders’ feelings of not being sure about the direction, and outcomes, of the initiative and their roles in the initiative. While uncertainty among stakeholders regarding their tasks and deliverables is common in multi-stakeholder initiatives (Reypens et al., 2019) as well as uncertainty regarding project outcomes in circular economy projects (Geissdoerfer et al., 2017), our study showed that involving local communities increased the relative importance and role of uncertainty. This was primarily caused by the fact that local community members did not have a clear understanding of the circular economy, whereas other stakeholders also did not know what the circular economy could mean for local community members. Therefore, the involved stakeholders could not predict the direction and outcomes of the initiative as these were largely dependent on the input of the local community members. Our findings highlighted that this could lead to a lack of inclusiveness such as instances in which local community members were reluctant to collaborate as they were not certain about the outcomes of the initiative.

To deal with uncertainty, we found that there was a need to first interact with local community members on the topic of the circular economy to build their knowledge and skills. Our study showed that this could be achieved through the addition of an ‘exploration phase’, in which local communities were enabled to explore what the circular economy could mean in their environment. Our study showed that the ‘exploration phase’ could assist in addressing uncertainty among the stakeholders as it increased the awareness of the involved stakeholders about the perspectives and potential roles of local communities in circular economy approaches. This is important, as our study highlighted that not all involved stakeholders may initially perceive local community involvement as relevant for circular economy approaches. The ‘exploration phase’ was also adopted as a strategy for ‘levelling the playing field’, enabling marginalized stakeholders to have a voice and increase trust (Gray & Purdy, 2018; Purdy, 2012).

However, our results also indicated that the ‘exploration phase’ did not prevent conflicts related to uncertainty entirely. Uncertainty remained an important theme during the initiative and the involved stakeholders expressed uncertainty about their roles in, and outcomes of, the initiative during all phases. These results show that in multi-stakeholder initiatives which involve marginalized stakeholders, conflicts surrounding uncertainty may not be easily solved. Instead, it may be necessary to create a situation in which uncertainty is acceptable for the involved stakeholders. Our study showed that this could for example be achieved by framing the initiative as an experiment. Furthermore, the aim should not be to eliminate uncertainty entirely as our study highlighted that a certain level of uncertainty, especially at the start of the initiative, could be beneficial in order to allow for the exploration and inclusion of unexpected community perspectives, thus increasing their ability to have a voice in the initiative.

### Disagreement

In line with recent multi-stakeholder initiative literature (e.g. Hovring et al., 2018; Reypens et al., 2019), our results indicated that there were multiple and diverse understandings of the circular economy among the involved stakeholders, which led to disagreement. Our study highlighted that local community involvement added an additional, more socially oriented, understanding of the goals of circular economy approaches which made generating shared approaches even harder. Furthermore, in line with Roloff (2008), we found that including or excluding local community stakeholders and their perspectives was a sensitive issue, especially in the initial phases of the initiative. Our results highlighted that, while it may be beneficial to start with a smaller stakeholder group to enable efficient communication (Roloff, 2008), this may lead to disappointment among local communities and a reluctance to collaborate in later phases of the initiative, thus limiting inclusiveness. 

While issue-focussed stakeholder management aims to overcome initial disagreement and generate shared perspectives (Roloff, 2008), we found that this was not always achieved in our case. While disagreement is frequently framed as undesirable (Brand et al., 2019), our study showed that disagreement could also lead to creative solutions. For instance, because they did not agree on the goals of the circular economy approaches, the involved stakeholders tried to come up with circular economy approaches that could satisfy multiple goals simultaneously. Our findings indicated that shared understanding of the goals of circular economy approaches may thus not be necessary in order to generate shared circular economy approaches. This refers to a weak form of consensus where stakeholders do not hold the same beliefs and values regarding an issue but agree on a course of action (Brand et al., 2019). Furthermore, conflicts such as disagreement can be useful to identify critical perspectives on the circular economy and help prevent false consensus (Brown & Dillard, 2013). This is important as an emphasis on consensus likely masks differences in perspectives and can limit the input of disadvantaged groups. However, it is important to note that high levels of disagreement may also have negative effects. Our study showed for instance that, due to their disagreement on the goals of the circular economy approaches, stakeholders may not feel ownership over and put effort in shared circular economy approaches.

### Consensus- and Domination-Based Management

Our results showed that inequality regarding knowledge about the circular economy and the resources of local communities and other stakeholders may hinder the involvement of local communities in multi-stakeholder initiatives. Our study highlighted that domination-based strategies to manage multi-stakeholder initiatives may be adopted to reduce inequality. We refer to domination-based strategies as strategies in which one actor sets the agenda, decides which stakeholders to include and steers interactions, in contrast to consensus-based strategies where the involved stakeholders collectively negotiate the agenda and openly share their perspectives. While Roloff (2008) previously argued that no single organization can or should be in control of the issue-focussed stakeholder management process, our study highlighted that the housing association did adopt domination-based strategies to keep some level of control and ensure that the perspectives of local community members were taken into account, suggesting that these strategies may bring advantages at certain points. This finding is in line with Reypens et al. (2019) who argue that, when dealing with many stakeholders, domination-based strategies may be more effective compared to consensus-based strategies. Our results showed that within contexts where marginalized stakeholders are involved, the adoption of domination-based strategies may be necessary to deal with knowledge and resource differences, safeguard equality and ensure beneficial outcomes for these stakeholders. Our results also highlighted the important role organizations with a close proximity to local communities, such as housing associations, can play in this regard. These organizations can, through steering relationships and coordinating exploration efforts, reduce the barriers to local community involvement and bring relevant stakeholders together in an equal setting for the benefits of local communities.

Our results also highlighted the importance of combining domination-based strategies with consensus-based strategies to allow for the emergence of new interactions and solutions. This is important as our findings showed that domination-based strategies may also have negative outcomes for local communities, for example when leading stakeholders prespecified topics for discussion and in this way limit the room for input from local communities. An emphasis on consensus-based strategies may thus lead to inequality, whereas an extensive focus on domination-based strategies may lead to limited room for new input from local communities and a lack of creative solutions. The combination of domination- and consensus-based strategies can be a challenging task for organizations as they have to decide on the right balance which can lead to conflicts due to different opinions among employees. Our results showed that conflicts about which strategy to adopt were not only apparent at the start of the initiative, but resurfaced, for instance when organizing the acquaintance and agreement phases. A constant reflection on the right balance between domination- and consensus-based management strategies may thus be needed, taking into account the different phases of the initiative. Our results indicated for example that it may be important to adopt more consensus-based management strategies in the first phases of the initiative to allow for the inclusion of local community perspectives, while in later phases it may be beneficial to shift to more domination-based strategies in order to protect the interests of local community members and ensure beneficial outcomes.

### Conclusion: Creating a Balance to Involve Local Communities in Multi-stakeholder Initiatives

The involvement of marginalized stakeholders, and of local communities in particular, in multi-stakeholder initiatives is an under-explored area of research (Fransen & Kolk, 2007; Khazaei et al., 2015). While the benefits of involving local communities are acknowledged, such as the enhanced legitimization of decision-making processes and joint learning, it has also been shown that these benefits are often not realized in multi-stakeholder initiatives due to a lack of both inclusiveness and procedural fairness (Cheyns & Riisgaard, 2014; Easter et al., 2022; Fransen & Kolk, 2007). Our in-depth action research study of a circular neighbourhood initiative highlights several aspects that can improve our understanding of how to successfully involve marginalized stakeholders, such as local communities.

First, our study highlighted that neither the inclusion of marginalized stakeholders nor procedural fairness can be ensured at the start of multi-stakeholder initiatives (Fransen & Kolk, 2007). Our results indicated that, even when marginalized stakeholder inclusion is addressed at the start of an initiative, their perspectives can be disregarded because other more powerful representatives, for example from business, dominate the discussion. In fact, our results showed that continuous management of three factors over the course of the initiative is needed, including uncertainty, disagreement and consensus- vs. domination-based management strategies. By uncovering these dynamics, our results add to the multi-stakeholder initiative literature (Baumann-Pauly et al., 2017; Cheyns & Riisgaard, 2014; Easter et al., 2022; Fransen & Kolk, 2007) by offering in-depth insights into how and which factors can be managed during the course of multi-stakeholder initiatives to reduce unethical behaviours, such as the exclusion of social issues form the dialogue and the systematic favour of some actors over others.

Second, and in line with tentative suggestions made by previous research (Gray & Purdy, 2018), our study contributes to the multi-stakeholder initiative literature (Easter et al., 2022; Gray & Purdy, 2018; Mena & Palazzo, 2012; Roloff, 2008; Zeyen et al., 2016) by showing that factors often treated as challenges can in fact play a beneficial role in these initiatives. In particular, our study showed that uncertainty and disagreement can play an important role, for instance by enabling the inclusion of unexpected local community perspectives. Furthermore, we add to previous insights by highlighting that multi-stakeholder initiatives may need to go beyond a choice between consensus-based or domination-based strategies (Reypens et al., 2019; Roloff, 2008). Instead, our findings show the importance of combining both strategies, as both consensus- and domination-based strategies have advantages at different phases of multi-stakeholder initiatives that aim to involve marginalized stakeholders. This can cause changes in the relationships between actors in the initiative over time, where some actors may be central at certain stages during the initiative while during other stages no central actor may be present, improving inclusion for marginalized stakeholders.

**Fig. 5:**
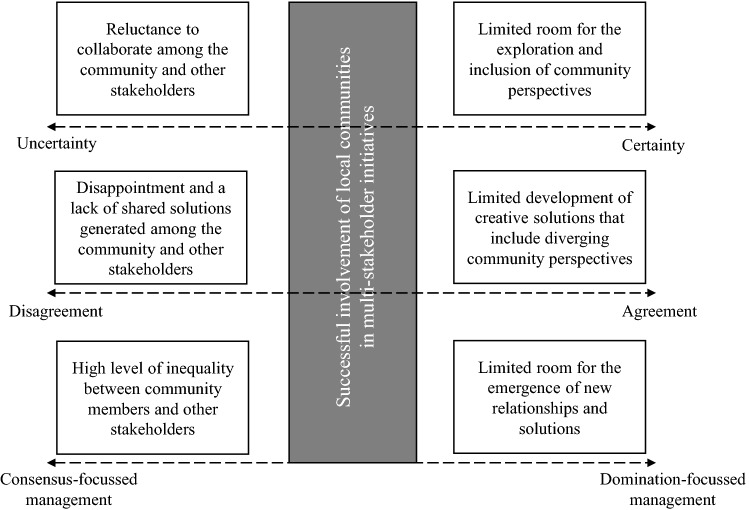
Balancing three factors to involve local communities in multi-stakeholder initiatives

**Fig. 6:**
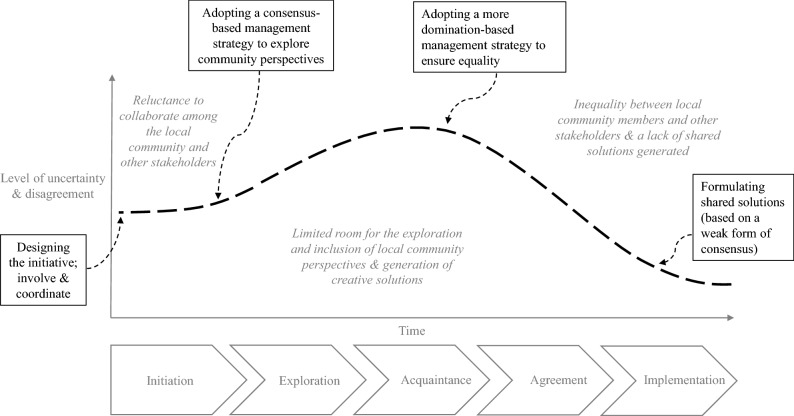
A process perspective on involving local communities in multi-stakeholder initiatives

Combining these insights, our results contribute to the multi-stakeholder initiative literature (Cheyns & Riisgaard, 2014; Easter et al., 2022; Fransen & Kolk, 2007; Gray & Purdy, 2018; Reypens et al., 2019) by establishing that multi-stakeholder initiatives require a constant reflection on and management of a balance between uncertainty–certainty, disagreement–agreement and domination- and consensus-based management in order to successfully include marginalized stakeholders. Figure 5 shows the need for balancing these aspects by highlighting the potentially negative implications of high levels of disagreement, uncertainty and consensus-based management, while showing that high levels of certainty, agreement and domination-based management in these initiatives can also lead to negative outcomes. Our findings highlight that this balance can be struck by taking a temporally sensitive approach, moving between agreement–disagreement and certainty–uncertainty by adopting both consensus-based and domination-based strategies at different phases of the initiative. These insights add to the literature on multi-stakeholder initiatives (Baumann-Pauly et al., 2017; Fransen & Kolk, 2007; Moog et al., 2015; Palazzo & Scherer, 2006), by showing that the successful involvement of marginalized stakeholders requires different strategies during different phases of a multi-stakeholder initiative. Figure 6 highlights that adopting a domination-based strategy can be useful at the very start of the initiative, to provide for a coordinated exploration of local community perspectives, as the high level of insecurity and ambiguity may otherwise discourage stakeholders to become involved. Subsequently, adopting a consensus-based management strategy, and thus allowing for higher levels of insecurity and disagreement, during the exploration and acquaintance phases can be beneficial to create enough room for the exploration of local community perspectives and generation of creative solutions. Finally, switching to domination-based strategies, creating more certainty and agreement, may be needed in the agreement and implementation phases in order to ensure equality and generate shared solutions. Instead of viewing these solutions as a rationally achieved form of consensus (Mena & Palazzo, 2012), we see them in line with a weak form of consensus (Brand et al., 2019), where the involved stakeholders do not necessarily hold the same beliefs and values regarding the central topic (e.g. circular economy) but can agree on a course of action based on certain principles (e.g. the desire to create a pleasant neighbourhood). This is in line with recent research (Arenas et al., 2020), which has suggested that the focus in multi-stakeholder initiatives should be on meta-consensus, a basic agreement about some fundamental principles that can facilitate ongoing contestation and deliberation. By taking a temporally sensitive approach, diverse key aspects for the involvement of marginalized stakeholders that have been highlighted by previous research can thus be addressed simultaneously, including assigning a clear role for marginalized stakeholders at the start of the initiative (Fransen & Kolk, 2007), creating room for minority perspectives and contestation (Banerjee, 2008; Cheyns & Riisgaard, 2014; Moog et al., 2015) and generating shared solutions (Easter et al., 2022; Gray & Purdy, 2018). Furthermore, taking a temporality sensitive approach may be an important way to deal with the seemingly conflicting situation inherent in multi-stakeholder initiatives, where disagreement and uncertainty are necessary for inclusiveness (Nanz & Steffek, 2005), but (in case these factors are too strong) may simultaneously reduce effectiveness and even lead to the collapse of deliberation (Arenas et al., 2020; Gray & Purdy, 2018). However, striking the right balance is complex and can be easily disrupted due to exogenous shocks which may shift the priorities of the involved stakeholders. Furthermore, the balance can easily tip over to one side and could for example, in cases of high levels of disagreement, lead to a lack of perceived ownership of the proposed approaches among the involved stakeholders.

Our study also contributes to the circular economy literature by responding to calls for a wider recognition of social and ethical issues in the circular economy (Geissdoerfer et al., 2017; Inigo & Blok, 2019; Kristensen & Mosgaard, 2020; Murray et al., 2017). Definitions of the circular economy are often ambiguous in both research and practice (Kirchherr et al., 2017). Our study confirmed this ambiguity, showing that the diverse stakeholders involved in the initiative held different understandings of the circular economy, including a more socially oriented perspective that has not been acknowledged in previous literature (Kirchherr et al., 2017). In doing so, our study also highlights that the involvement of local communities is an important ethical consideration which can assist in including previously neglected perspectives in the dialogue. We also contribute to the circular economy literature (e.g. Hobson & Lynch, 2016) by showing that local communities can play a bigger role in the circular economy than merely as consumers having to accept or reject new business models. We find that local communities can act as co-creators of circular strategies, which can result in non-monetary approaches that adhere to local norms and values, such as local sharing initiatives.

### Limitations and Future Research

There were several limitations in this study, which point to areas for future research. First, this was an exploratory study focussing on a single initiative. Therefore, the results are context specific. For example, uncertainty and disagreement may be more apparent in the circular economy context, due to the ambiguity surrounding the concept. Therefore, the involvement of communities may work differently in multi-stakeholder initiatives in which other issues, especially those closer to local communities, such as health and food, are addressed. While future research would be needed to confirm the relevance of our results, we believe that our findings will likely offer some value in these contexts as there also power imbalances and marginalized stakeholders are involved. Furthermore, this research focussed on a local multi-stakeholder initiative for problem solving and idea generation on a community level. Involving communities in industry-specific or global multi-stakeholder initiatives (Roloff, 2008) may have different implications, for example intensifying challenges related to selecting community members and ensuring the relevance of the initiative to community members. Further research is therefore needed to explore community involvement in other types of multi-stakeholder initiatives. Additionally, we focussed in this research on communities of place in the Netherlands. Therefore, the involvement of communities characterized by interaction or identity, such as communities of interest, remains unexplored. Additional research is also needed to focus on the involvement of communities in other countries and cultures. Power distance is for instance relatively low in the Netherlands, making managers and policy officials more open to collaborate with those lower in the hierarchy, such as employees and communities (Ringov & Zollo, 2007). More extensive challenges with community involvement may be experienced in countries with higher power distance. Our results are likely to offer some assistance on how to manage these challenges. However, the specific timing of strategic mixes as well as the degree of, for instance, domination in different contexts are questions that future research should explore. Furthermore, the involvement of the housing association may provide an exceptional context due to the close proximity of the housing association to the local community. While this context provided relevant insights, questions arise about community involvement in multi-stakeholder initiatives where initial interaction with communities is limited.

Second, our research assisted in identifying multiple challenges that have to be addressed in the involvement of communities in multi-stakeholder initiatives. Our study highlighted the importance of striking a balance between agreement–disagreement, certainty–uncertainty and consensus- and domination-based management. Further research is needed to develop and assess guidelines specifying how organizations and policymakers can create this balance, which was beyond the empirical scope of our research. Furthermore, our study highlighted that domination-based strategies to manage multi-stakeholder initiatives could bring several advantages. However, this also raises questions about how and by whom appropriate decisions can be made in this regard. Future studies are therefore needed to investigate these considerations and explore which organizations can best manage the involvement of communities in multi-stakeholder initiatives. Additionally, there is still a need for the exploration of differences within communities, for example by investigating how different community groups could be best involved.

Third, the nature of action research and our approach has implications for the limitations of the research. Our research impacted the involved stakeholders; however, the impact was limited due to our research design. Our goals were to support the design and execution of the multi-stakeholder initiative and evaluate the involvement of communities. At the start of the research, it was unclear how circular economy approaches could be implemented in the neighbourhood and how communities could be involved. Upon the completion of this research, new ways to involve the community and implement circular economy approaches were discovered. However, the lasting value of these insights and the actual implementation of circular economy approaches in the neighbourhood is unknown. Therefore, future research could adopt more longitudinal designs to investigate the lasting impact of action research approaches. Furthermore, questions about how researchers can contribute effectively to multi-stakeholder initiatives through action research approaches remain unanswered. Additionally, in our action research approach we were closely involved with the activities of the housing association, relating to their perspectives on community involvement which may have impacted our results. In the future, research could therefore explore community involvement from the perspectives of other stakeholders. Furthermore, in our data collection we focussed on those community members that played pro-active roles in the community and/or were able and willing to participate in the initiative. Therefore, the perspectives of some vulnerable groups may have been missed. Our data analysis also involves limitations, as we built our research on pre-existing concepts and involved the stakeholders in selecting emerging themes. Adopting inductive approaches in the data analysis of future studies may result in the identification of new themes that may not have appeared in our analysis.

Fourth, due to the COVID-19 pandemic we were not able to explore the direct outcomes of the initiative as it was terminated before the circular economy approaches were implemented. More research is therefore needed to explore the circular economy approaches that can result from involving communities in multi-stakeholder initiatives, including their effects. Additionally, questions arise about the impact of early project termination, especially in terms of the organization of future initiatives which may have to deal with increased reluctance of marginalized stakeholders to collaborate. Researchers may explore these questions by, for example, investigating if and how early termination can influence the tolerance of the involved stakeholders for uncertainty in future initiatives. Furthermore, this research did not explore all the phases of the issue-focussed stakeholder management process. We were not able to explore the continuation, institutionalization and extinction phases, meaning future works is necessary to explore these phases, as they may assist in creating lasting relationships with communities. In addition, questions arise about how multi-stakeholder initiatives involving marginalized stakeholders can deal with exogenous shocks, such as the COVID-19 pandemic. As such shocks may become more apparent, future work exploring how marginalized stakeholder involvement can be maintained during such events is important.

### Electronic supplementary material

Below is the link to the electronic supplementary material.

Supplementary file1 (PDF 349 KB)

Supplementary file2 (PDF 105 KB)

## Appendix

### Appendix A: Interview Protocol, Interviews During the Diagnosing Step

#### Interview Goals

Gain insights into the neighbourhood, the action plan being designed for the neighbourhood, the potential for circular economy approaches in the neighbourhood and the potential for community involvement in the design and implementation of circular economy approaches in the neighbourhood

#### Sub-Goals

Gain insights in the following:

- The neighbourhood, including its challenges and opportunities.
- The action plan being designed for the neighbourhood.
- The different stakeholders involved in the neighbourhood and the action plan.
- The understandings of the involved stakeholders on the circular economy and the adoption of circular economy approaches in the neighbourhood.
- The perspectives of the involved stakeholders on the involvement of the community in the design and implementation of circular economy approaches in the neighbourhood.

#### Interview Questions

1. Introduction of the organization and interviewee:
  - Can you shortly introduce your organization and function?
  - What is/has been your role in the action plan being designed for the neighbourhood?
2. Exploring the neighbourhood and action plan:
  - What are, in your opinion, challenges in the neighbourhood?
  - What are, in your opinion, opportunities in the neighbourhood?
  - What are, in your opinion, ways in which these challenges and opportunities can be addressed in the action plan for the neighbourhood?
  - How is the action plan for the neighbourhood being developed?
  - What are the focus points in the action plan for the neighbourhood?
3. Exploring the topic of the circular economy:
  - Are you familiar with the concept of the circular economy? If yes, how would you define the circular economy? (if not, the definition of the circular economy by Kirchherr et al. (2017) is discussed)
  - Are you involved in any projects or activities around the circular economy?
4. Exploring the potential for circular economy approaches in the neighbourhood:
  - Do you think circular economy approaches could be implemented in the neighbourhood and the action plan? If not, why not? If yes, how do you think it could be implemented?
  - Do you think circular economy approaches could be valuable for the neighbourhood? If not, why not? If yes, why and how?
  - If circular economy approaches would be implemented in the neighbourhood, how and where should it, in your opinion, be implemented?
  - What do you think would be challenges of adopting circular economy approaches in the neighbourhood?
  - What do you think would be opportunities for adopting circular economy approaches in the neighbourhood?
5. Exploring the potential for community involvement:
  - Do you think that the community should be involved in the design and implementation of circular economy approaches in the neighbourhood? If yes, why? If not, why not?
  - How do you think the community could be involved in the design and implementation of circular economy approaches in the neighbourhood?
  - What do you think would be the outcomes of involving the community in the design and implementation of circular economy approaches in the neighbourhood?
  - What do you think would be challenges of involving the community in the design and implementation of circular economy approaches in the neighbourhood?
  - What do you think would be opportunities of involving the community in the design and implementation of circular economy approaches in the neighbourhood?

### Appendix B: Interview Protocol, Interviews During the Evaluation Step

#### Interview Goals

Gain insights into the experiences during and opinions on the discussion meetings of the involved stakeholders

#### Sub-Goals

Gain insights in the following:

- The involved stakeholders and their reasons for participating in the discussion meetings.
- The experiences of the involved stakeholders during the discussion meetings.
- The evaluation of the involved stakeholders on the discussion meetings, and the involvement of community members in these meetings in particular.

#### Interview Questions

1. Introduction of the organization and interviewee:
  - Can you shortly introduce your organization and function?
  - What was you reason for participating in the discussion meetings?
  - What were your expectations of the discussion meetings?
2. Exploring the experiences of the interviewee during the discussion meetings:
  - How did you experience the discussion meetings? Was it a positive or negative experience?
  - How did you experience working together with other stakeholders during the discussion meetings? Was it easy or difficult to discuss with the others?
  - What were challenges you experienced during the discussion meetings? Were you able to deal with these challenges?
  - What were opportunities you experienced during the discussion meetings?
3. Exploring the evaluations of the stakeholders on the discussion meetings:
  - Did you learn anything from the discussion meetings? If yes, what did you learn? If not, why not?
  - Are you happy with how the discussion meetings went? If yes, why? If not, why not?
  - Did your perspective on the adoption of circular economy approaches in the neighbourhood change as a result of the discussion meetings? If yes, how did it change? If not, why not?
  - Do you think the involvement of community members in the discussion meetings was valuable for the design and implementation of circular economy approaches in the neighbourhood? If yes, why? If not, why not?
