# Supplementary material for: Breaking the Cycle of Marginalization: How to Involve Local Communities in Multi-stakeholder Initiatives?
Source: J Bus Ethics. 2022 Sep 22:1–32. Online ahead of print. doi: 10.1007/s10551-022-05252-5 (PMC9510282; doi:10.1007/s10551-022-05252-5)
Supplement: Supplementary file 1 — Supplementary file1 (PDF 349 KB) [file 10551_2022_5252_MOESM1_ESM.pdf]

Appendix C: illustration of the workshop at the primary school and discussion regarding reuse and repair

In this vignette the workshop at the primary school and the stakeholder discussion for one of the included topics, reuse and repair, will be outlined.

Storyboard created by one of the groups

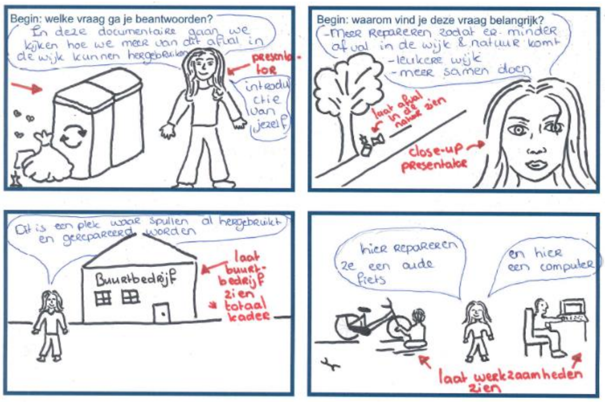

Three teams of primary school children collected insights on the topic of reuse and repair in their neighbourhood. All three groups were supervised by two bachelor students from the University. With the help of the bachelor students the children first designed a plan for their documentary in a short storyboard.

After finishing their storyboards, the groups ventured into the neighbourhood and visited several community organizations including the neighbourhood company, the local second-hand clothing store and local bicycle shop. The children interviewed these organizations, asking questions about what they were doing in terms of reuse and repair and if they had new ideas for reuse and repair in the neighbourhood. Furthermore, the groups interviewed community members on the street and at local stores to ask them their opinion about reuse and repair at home and in the neighbourhood. The groups included several insights in their documentaries, such as:

Groups going into the neighbourhood and editing their documentaries

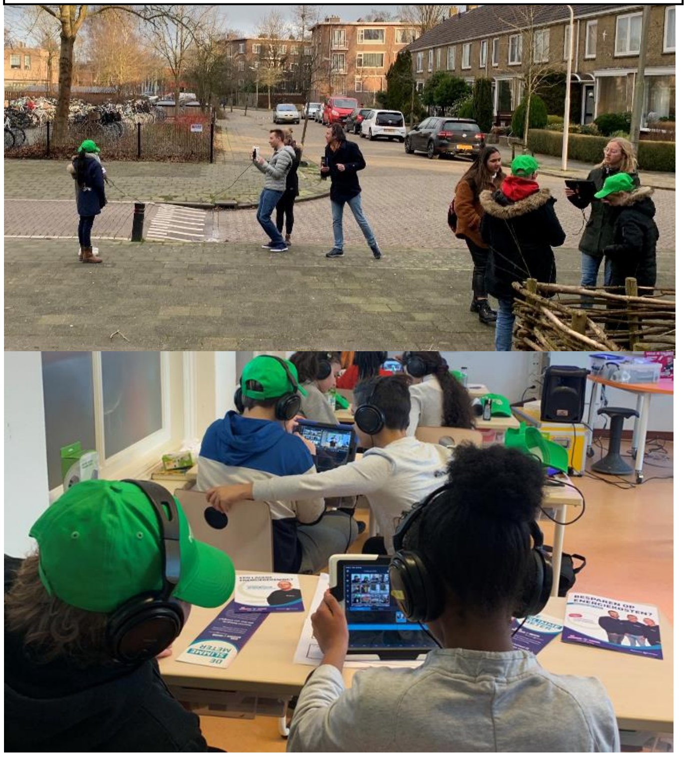

- The neighbourhood company repaired a lot of broken things of people in the neighbourhood, such as bicycles and small appliances.
- People in the neighbourhood that were interviewed by the groups thought it was important to repair broken appliances, however they were not always able to do this themselves.
- People in the neighbourhood that were interviewed by the groups were positive about reuse and already tried to reuse things, such as plastic bags and bottles.
- Sharing could help people in the neighbourhood with a low income and enabled them to get a job, for example at the second-hand store.
- People in the neighbourhood that were interviewed by the groups though the separation of waste and potential for reuse could be improved as they had seen this was arranged better in other areas.

After filming their documentaries, the groups came back to school in order to edit them. At the end of the workshop the documentaries of the students were shown in a premiere at the school including all students of the school, their parents and several stakeholders in the neighbourhood.

Two weeks after the premiere at the primary school the first discussion meetings took place. One group was formed around the topic of reuse and repair including two community members, the owner of the second-hand store, an employee of the neighbourhood company, the housing association social affairs project leader, the municipality district manager and the strategy manager of the builder. During this discussion meeting the group thought about how reuse and repair could be improved in the neighbourhood. The documentaries and the insights of the primary school students were used at the start of the first discussion meeting. The discussion groups got several materials, including maps of the neighbourhood and small blocks to visualise their ideas.

Premiere of the documentaries at the primary school & the discussion meeting regarding reuse and repair

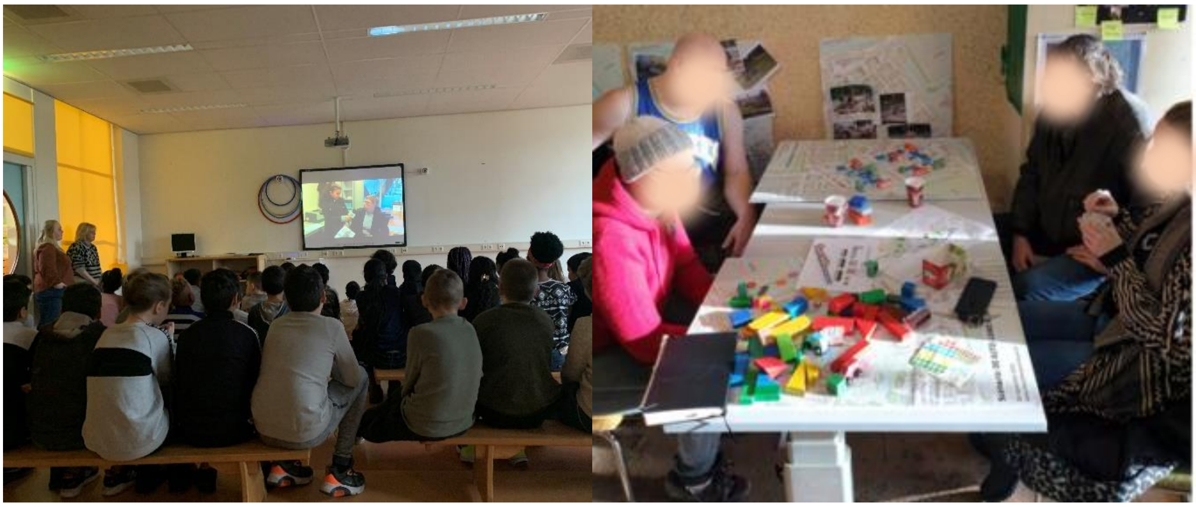

During the first hour of the first discussion meeting the involved community members and community organizations were talking more compared to the other involved stakeholders, relating to the findings of the students. They discussed for instance if they recognized that the potential for reuse could be improved and, together with the other stakeholders, thought about new solutions such as small local libraries or other second-hand stores in the neighbourhood. After the break, the conversation moved towards the reuse of materials. During this hour the conversation was mainly led by the builder and municipality district manager, who discussed the potential for the reuse of materials during the demolition and construction of houses in the neighbourhood for environmental gains. At the end of the discussion, several initial ideas for reuse and repair had been voiced but no general strategy had been formed.

The conversation on the reuse of materials continued in a second discussion meeting. The conversation did not always flow smoothly. For example, during this second meeting one of the community members initiated the idea to make demolition materials available to community members to build sheds in their gardens or shared libraries in the neighbourhood. However, the builder and municipality district manager directly voiced many arguments against this, such as safety and difficulties with transportation, dismissing the idea before the rest of the group could give their input. The housing association social affairs project leader intervened in the conversation by emphasizing the potential of reusing materials to also be of benefit to community members, next to the environmental gains. In the end of the second discussion meeting the group decided to focus on the reuse of materials in the demolition of houses and enable community members to use left-over materials for small at home projects in this process.
