# Supplementary material for: Breaking the Cycle of Marginalization: How to Involve Local Communities in Multi-stakeholder Initiatives?
Source: J Bus Ethics. 2022 Sep 22:1–32. Online ahead of print. doi: 10.1007/s10551-022-05252-5 (PMC9510282; doi:10.1007/s10551-022-05252-5)
Supplement: Supplementary file 2 — Supplementary file2 (PDF 105 KB) [file 10551_2022_5252_MOESM2_ESM.pdf]

## Supplementary material: data structure

1st-order concepts 2nd-order categories aggregate dimensions

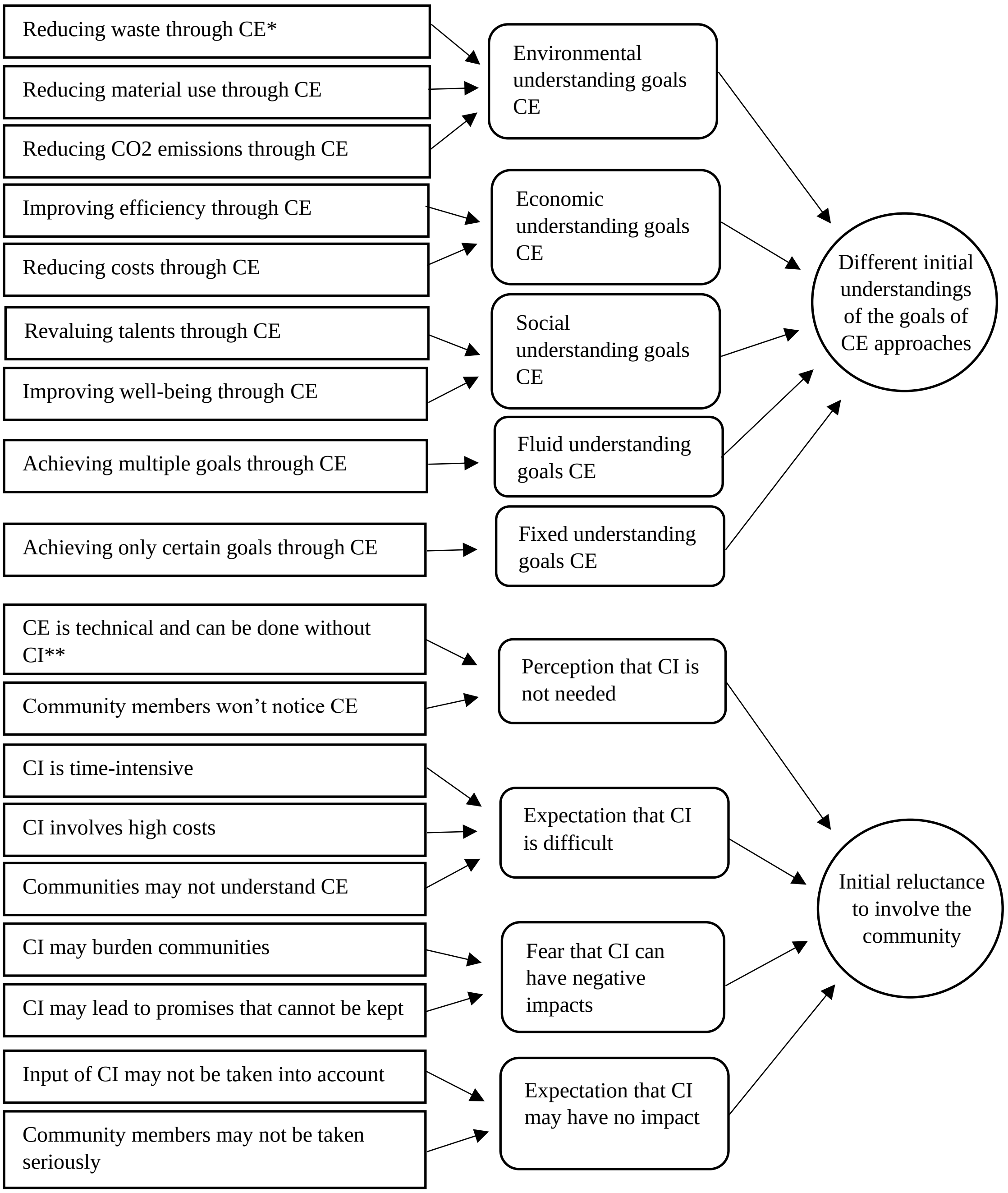


*CE: circular economy
** CI: community involvement

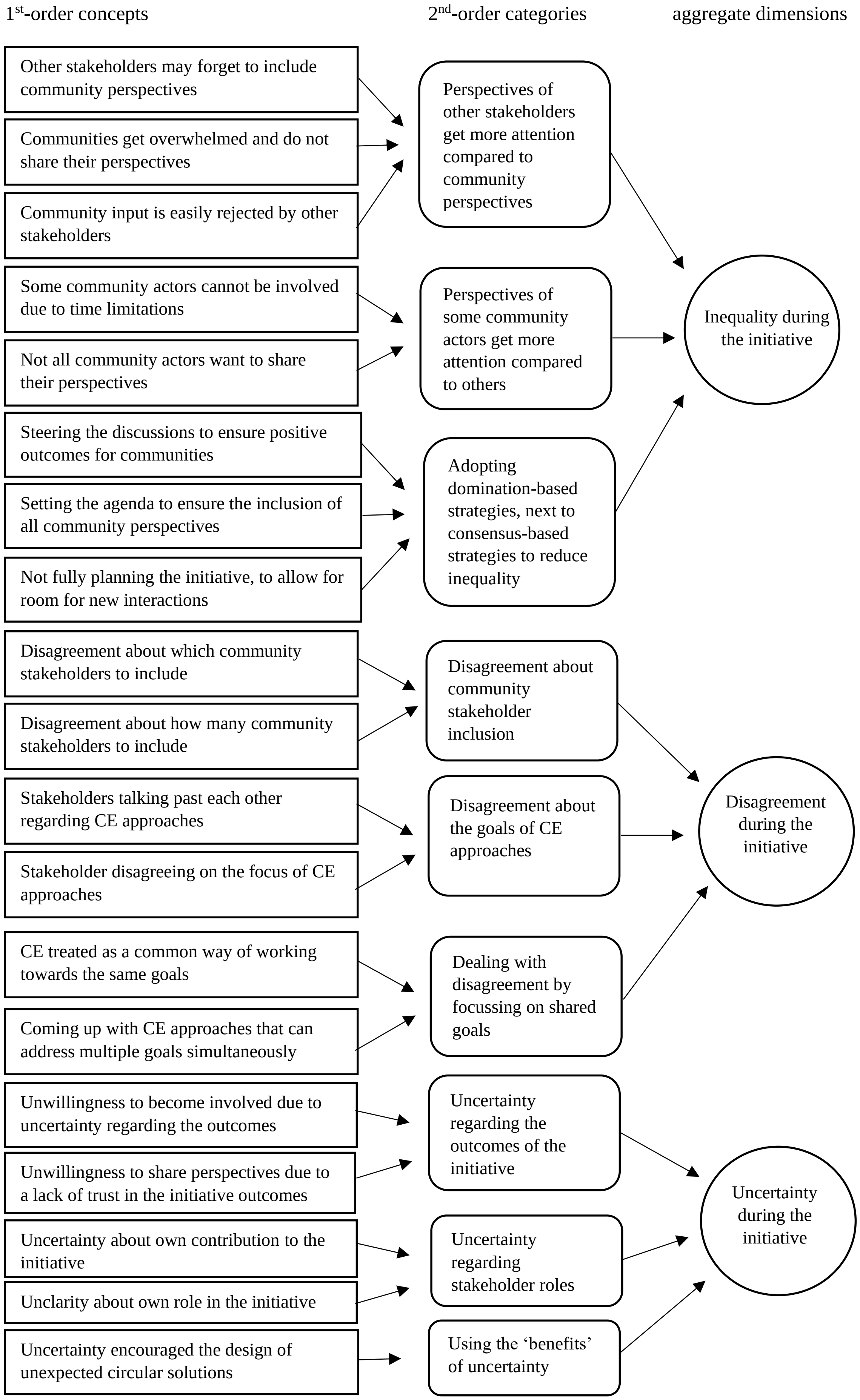

1st-order concepts
2nd-order categories
aggregate dimensions
Other stakeholders may forget to include community perspectives
Communities get overwhelmed and do not share their perspectives
Community input is easily rejected by other stakeholders
Some community actors cannot be involved due to time limitations
Not all community actors want to share their perspectives
Steering the discussions to ensure positive outcomes for communities
Setting the agenda to ensure the inclusion of all community perspectives
Not fully planning the initiative, to allow for room for new interactions
Disagreement about which community stakeholders to include
Disagreement about how many community stakeholders to include
Stakeholders talking past each other regarding CE approaches
Stakeholder disagreeing on the focus of CE approaches
CE treated as a common way of working towards the same goals
Coming up with CE approaches that can address multiple goals simultaneously
Unwillingness to become involved due to uncertainty regarding the outcomes
Unwillingness to share perspectives due to a lack of trust in the initiative outcomes
Uncertainty about own contribution to the initiative
Unclarity about own role in the initiative
Uncertainty encouraged the design of unexpected circular solutions
Perspectives of other stakeholders get more attention compared to community perspectives
Perspectives of some community actors get more attention compared to others
Adopting domination-based strategies, next to consensus-based strategies to reduce inequality
Disagreement about community stakeholder inclusion
Disagreement about the goals of CE approaches
Dealing with disagreement by focussing on shared goals
Uncertainty regarding the outcomes of the CE initiative
Uncertainty regarding stakeholder roles
Using the 'benefits' of uncertainty
Inequality during the initiative
Disagreement during the initiative
Uncertainty during the initiative

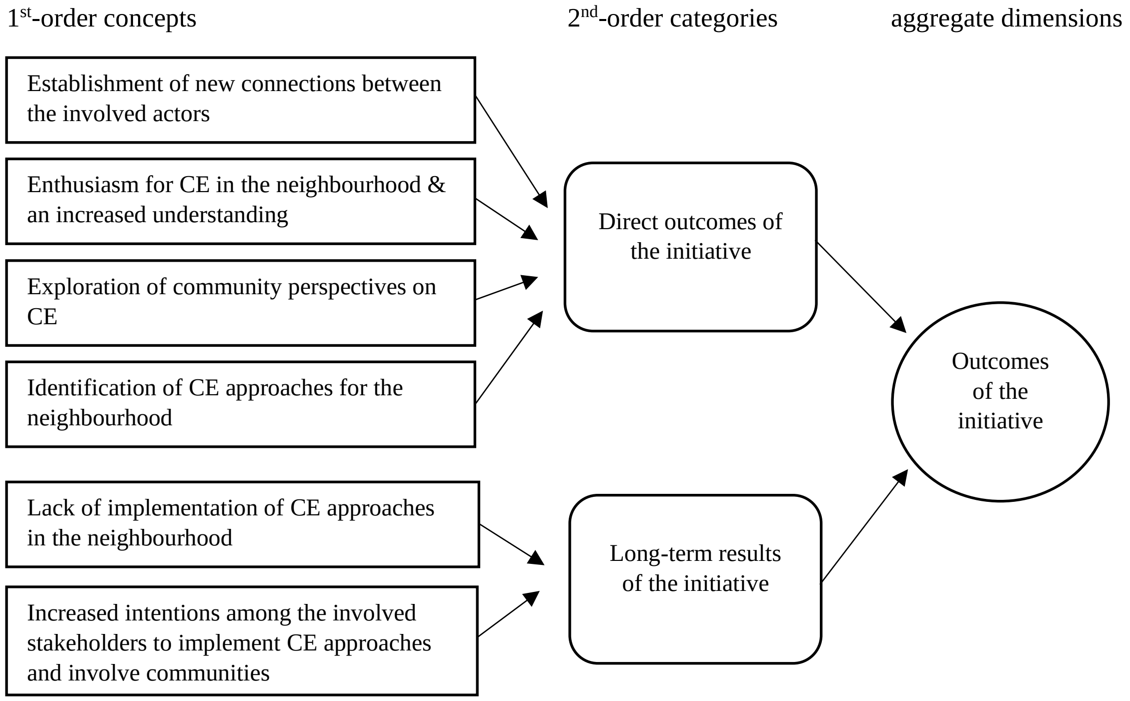

1st-order concepts
2nd-order categories
aggregate dimensions
Establishment of new connections between the involved actors
Enthusiasm for CE in the neighbourhood & an increased understanding
Exploration of community perspectives on CE
Identification of CE approaches for the neighbourhood
Lack of implementation of CE approaches in the neighbourhood
Increased intentions among the involved stakeholders to implement CE approaches and involve communities
Direct outcomes of the initiative
Long-term results of the initiative
Outcomes of the initiative
